# Multicyclic D‐Stereospecific Hydrolase Dimer With High Sustained Activity

**DOI:** 10.1002/anie.202521611

**Published:** 2026-03-20

**Authors:** Anissa Haim, Sandra Liebscher, Rasmus Klintrot, Lorenzo Vallino, Marcelo Masman, Andreas H. Simon, Marianne Hahn, Sven Hennig, Saskia Neubacher, Frank Bordusa, Tom N. Grossmann

**Affiliations:** ^1^ Department of Chemistry and Pharmaceutical Sciences VU University Amsterdam Amsterdam The Netherlands; ^2^ Institute of Biochemistry and Biotechnology, Charles Tanford Protein Center Martin‐Luther‐University Halle‐Wittenberg Halle Germany; ^3^ Incircular B.V. Amsterdam The Netherlands; ^4^ Institute of Organic and Biomolecular Chemistry Georg‐August‐Universität Göttingen Göttingen Germany

**Keywords:** bioconjugation, cross‐linking, enzymes, INCYPRO, macrocyclization, protein engineering

## Abstract

Enzymes are powerful catalysts for selective transformations but often suffer from limited stability under operational conditions such as elevated temperature or the presence of organic cosolvents. While sequence‐based strategies have been widely used to improve stability, chemical protein engineering enables modifications beyond the natural amino acid repertoire thereby offering complementary routes to tailor enzyme function and robustness. Here, we apply the in situ cyclization of proteins (INCYPRO) to a D‐stereospecific hydrolase with low intrinsic thermal stability. Site‐specific macrocyclization substantially improved resilience to heat and cosolvent stress. Unexpectedly, we discovered a cross‐linked protein dimer with enhanced activity and thermal stability. The complex structure was confirmed by x‐ray crystallography. Extending the INCYPRO approach, we engineered a multicyclic enzyme dimer with a total of four cross‐linking sites, which not only retained high activity under benign conditions but also outperformed the wild‐type under stress. Our findings establish protein macrocyclization as a versatile strategy to stabilize both monomeric and multimeric enzymes, providing a powerful route to robust biocatalysts.

## Graphical abstract

Protein macrocyclization was applied to a thermally unstable D‐stereospecific hydrolase using in situ cyclization of proteins (INCYPRO). This site‐specific cross‐linking approach enhanced the resistance of the enzyme to heat and cosolvents. A cross‐linked dimer with improved activity and stability was identified and structurally confirmed, demonstrating the effectiveness of macrocyclization for protein engineering.

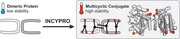

## 1 Introduction

Enzymes are capable of performing highly selective and efficient chemical transformations under aqueous and mild conditions. Their ability to act on complex, unprotected substrates often with high chemo‐ and stereoselectivity renders them attractive tools for a wide range of applications, including industrial biocatalysis and diagnostic analyte detection [1, 2, 3, 4]. However, the broader implementation of enzymes in non‐native and process‐relevant environments is often hindered by their limited stability [2, 3]. Enzymatic instability can manifest at multiple structural levels (i.e., secondary, tertiary and quaternary) posing significant challenges, particularly for enzymes that show a tendency for aggregation or function as complexes [5]. These structural vulnerabilities can lead to rapid loss of activity under operational conditions including elevated temperatures or in the presence of organic cosolvents. While traditional approaches to enhance enzyme robustness have focused on sequence‐based strategies such as directed evolution as well as computational or consensus‐based mutagenesis [6, 7, 8, 9, 10, 11], recent advances in chemical protein engineering have opened new avenues for stabilization [11, 12, 13, 14, 15, 16, 17, 18, 19, 20]. By introducing covalent modifications, non‐canonical amino acids, or synthetic cross‐links, chemical strategies offer complementary and often orthogonal means to tailor enzyme properties beyond the limits of natural amino acid diversity.

Protein macrocyclization has emerged as a particularly effective protein engineering approach for increasing protein stability [13, 21]. By covalently linking distant regions of a polypeptide chain, macrocyclization can reduce conformational entropy in the unfolded state, thereby increasing the thermodynamic stability of the folded protein [22]. Several macrocyclization techniques have been developed, including disulfide bond engineering, protein stapling via non‐canonical electrophilic amino acids, and the incorporation of synthetic linkers via bioorthogonal chemistry [11, 12, 13, 14, 23, 24, 25, 26, 27]. We have recently introduced the in situ cyclization of proteins (INCYPRO), which employs site‐selective incorporation of cysteine residues followed by chemoselective cross‐linking using hydrophilic trifunctional electrophiles [12, 28, 29, 30]. This approach enables the formation of covalent bridges across structurally distant sites, effectively locking the protein into its functional fold without compromising essential structural dynamics. INCYPRO has been shown to enhance protein stability toward thermal and chemical stress, while also reducing the tendency for aggregation and eventually inactivation [12, 30]. It has already been used for the stabilization of protein quaternary structures [30], however, only for trimeric complexes tailored to the C3 symmetry of applied cross‐linking reagents.

Herein, we report the chemical engineering of a D‐stereospecific hydrolase (DHy) with low thermal stability and high sensitivity toward organic cosolvents. To address these limitations, we introduced site‐specific cross‐links via INCYPRO leading to improved thermal resilience. Unexpectedly, we discovered that the enzyme could also be cross‐linked in a homodimeric form, resulting in a covalently stabilized dimer with significantly enhanced thermal stability. The dimeric structure was confirmed by x‐ray crystallography. Building on this observation, we engineered a highly active multicyclic dimeric variant by combining two orthogonal cross‐linking sites which exhibited superior performance under thermal and chemical stress.

## 2 Results and Discussion

### 2.1 Design, Cross‐Linking and Thermal Stability

**FIGURE 1:**
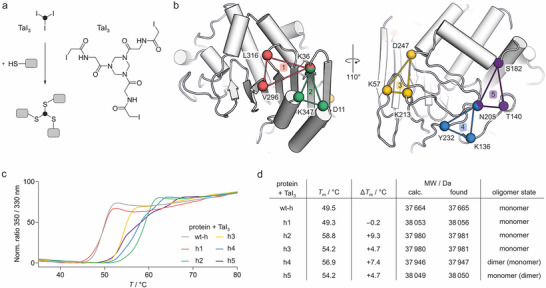
INCYPRO designs and stabilization: (a) INCYPRO‐based protein cross‐linking with iodoacetamide‐based triselectrophile TaI_3_. (b) Selected triple‐cysteine sites for INCYPRO resulting in variants h1–h5 (PDB ID 9spl, for sequences see Figure S1). (c) Thermal denaturation curves of wt‐h and variants h1–h5 after incubation with TaI_3_ using DSF as readout (*c*_protein_ = 50 µM, based on monomer, buffer: 50 mM HEPES (4‐(2‐hydroxyethyl)‐1‐piperazineethanesulfonic acid) pH 8.0, 50 mM NaCl). For thermal denaturation curves of uncross‐linked variants see Figures S2–S7. (d) Summary of melting temperatures and ESI‐TOF MS characterization (for procedure see Supporting Information). For mass spectra and list of signals see Figures S8–S13.

D‐stereospecific hydrolases recognize peptide and protein substrates with D‐configured amino acids and hydrolyze the corresponding amide [31, 32, 33, 34, 35]. We have reported the first example of such a hydrolase, DHy1 from *Bacillus thuringiensis*, capable of cleaving after D‐lysine and D‐arginine [31]. Since the wild‐type form of DHy1 (wt‐h) shows low thermal stability and sensitivity toward organic cosolvents, we considered the use of INCYPRO to address these limitations. INCYPRO requires the presence of three spatially aligned and accessible cysteines suitable for cross‐linking with triselectrophilic agents, such as the iodoacetamide‐modified triazinane TaI_3_ (Figure 1a) [28]. Based on the crystal structure of wt‐h (PDB ID 9spl), we selected five potentially suitable INCYPRO cross‐linking sites each involving the introduction of three cysteines (Figures 1b and S1). The design aimed for variants with Cα–Cα distances between the cysteines of 7–14 Å and at least *i*, *i*+20 spacing within the primary sequence (Table S1). The five triple‐cysteine variants (h1–h5) and wt‐h were expressed heterologously from *E. coli* and purified in analogy to earlier described protocols. Enzyme identity and purity were confirmed by sodium dodecyl sulfate‐polyacrylamide gel electrophoresis (SDS PAGE) and high‐performance liquid chromatography coupled with mass spectrometry (HPLC/MS, Figures S2–S7) [36, 37].

For cross‐linking, enzymes (*c *= 50 µM) were incubated with TaI_3_ (*c* = 300 µM) for 2 h in buffer (pH 8.0, 50 mM HEPES, 50 mM NaCl buffer). Thermal denaturation experiments were performed to determine melting temperatures (*T*_m_) using differential scanning fluorimetry (DSF) as readout (Figure 1c). For all triple‐cysteine variants (h1–h5), incubation with TaI_3_ resulted in increased thermal stability (Δ*T*_m_ > +4°C, Figures S2–S7). Overall, variants h2 and h4 showed the highest thermal stability after TaI_3_‐treatment with considerably increased stability compared to wild‐type enzyme wt‐h (Δ*T*_m_ = +9.3 and +7.4°C, respectively). Next, HPLC coupled with electrospray ionization time‐of‐flight mass spectrometry (TOF‐MS) was used to assess the degree of cross‐linking (Figure 1d). While wt‐h was not affected by TaI_3_‐treatment, we observed the disappearance of the unmodified protein for all variants (h1–h5). Based on HPLC/TOF‐MS, variants h1, h2, and h3 provided the expected fully cross‐linked product (Figures 1d and S3–S5). Interestingly, variants h4 and h5 exhibited both cross‐linked monomer and a mass corresponding to a protein dimer with two fully reacted cross‐links (h4_2_Ta_2_ and h5_2_Ta_2_). For these two variants, the presence of a covalently linked dimer after TaI_3_ treatment was also confirmed by SDS PAGE (Figures S6 and S7). In the case of h4, the dimer appeared to be the dominant species (Figures S6). Notably, both HPLC/TOF‐MS as well as SDS PAGE are performed under denaturing conditions confirming that the observed dimers are indeed covalently cross‐linked.

### 2.2 Isolation and Characterization of h2Ta, h4Ta, and h4_2_Ta_2_

**FIGURE 2:**
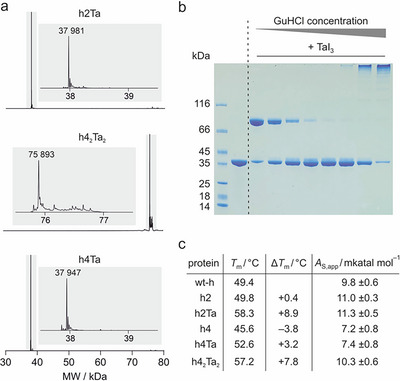
Cross‐linking of variants h2 and h4: (a) Deconvoluted TOF‐MS spectra of cross‐linked proteins h2Ta (calc. *MW* = 37980 Da), h4_2_Ta_2_ (calc. *MW* = 75892 Da) and h4Ta (calc. *MW* = 37946 Da). For TOF‐MS spectra and peak list see Figures S14, S16, and S17. (b) SDS PAGE showing cross‐linking of protein h4 with TaI_3_ in the presence of varying concentrations guanidine hydrochloride (GuHCl, *c* = 0, 0.1, 0.25, 0.5, 0.75, 1.0, 1.25 and 1.5 M). (c) Table with melting temperatures (*T*_m_, *c*_protein_ = 50 µM, based on monomer, buffer: 50 mM HEPES pH 8.0, 50 mM NaCl) and enzymatic activities (*A*_s,app_, *c*_protein_ = 0.5 µM, based on monomer, *c*_substrate_ = 50 µM, buffer: 100 mM phosphate, pH 8.0, 150 mM NaCl, errors = 1*σ*, *n* ≥ 3). For thermal denaturation curves see Figure S18.

In the initial cross‐linking experiments, variants h2 and h4 emerged as promising candidates due to their enhanced thermal stability. We were also interested in investigating the unexpected dimer formation of cross‐linked h4 in more detail. Initially, cross‐linked variant h2 (h2Ta) was obtained and the excess of TaI_3_ removed by size exclusion chromatography (SEC) providing the monomeric cross‐linked enzyme (Figure 2a, top). Next, cross‐linking of h4 was pursued aiming to obtain cross‐linked monomer h4Ta as well as dimer h4_2_Ta_2_, both of which occurred in the initial cross‐linking reaction (Figure S6). Initially, we tested cross‐linking at different protein concentrations observing increased dimer formation with increasing concentrations (Figure S15). Using a protein concentration of 200 and 500 µM TaI_3_, then indeed allowed the isolation of dimeric h4_2_Ta_2_ (Figure 2a, middle). We noticed that lowering protein concentration alone is not sufficient to obtain monomeric h4Ta and therefore considered the use of the chaotropic salt guanidine hydrochloride (GuHCl) to reduce h4 dimerization during cross‐linking. Indeed, increasing GuHCl concentrations up to 1 M resulted in decreased dimer formation as observed by SDS PAGE (Figure 2b). Above this concentration protein species occurred that would not migrate into the gel indicating protein polymerization due to unfolding. Using 0.75 M GuHCl during cross‐linking followed by SEC allowed the isolation of monomeric h4Ta (Figure 2a, bottom).

With wt‐h, h2, h4 and the three cross‐linked variants in hand, we performed thermal denaturation experiments to determine their stabilities (Figure 2c). Uncross‐linked triple‐cysteine variants h2 and h4 showed stabilities in the range of wt‐h (Δ*T*_m_ = +0.4 and –3.8°C, respectively, Figure 2c). As observed in the initial cross‐linking experiment, highest stability was obtained for h2Ta (*T*_m_ = 58.3°C). Interestingly, dimer h4_2_Ta_2_ showed higher thermal stability than monomer h4Ta (*T*_m_ = 57.2 vs. 52.6°C, Figure 2c), while originating from the same uncross‐linked precursor h4. Next, we assessed the activity (*A*_S,app_) of all enzymes under unstressed conditions (*T* = 30°C), using a peptide with a central D‐lysine as substrate (Figure S19) and ultra‐performance liquid chromatography (UPLC) as readout. Notably, all enzymes show activity with variant h2 as well as cross‐linked enzymes h2Ta and h4_2_Ta_2_ exceeding wt‐h activity (increased by 5%–15 %, Figure 2c). Variant h4 as well as monomeric h4Ta exhibit lower activity than wt‐h (decreased by ca. 15 %). Taken together, h2Ta and cross‐linked dimer h4_2_Ta_2_ exhibit highest thermal stability and enzymatic activities exceeding the wild‐type enzyme wt‐h.

### 2.3 Crystal Structure of h4_2_Ta_2_ Dimer

**FIGURE 3:**
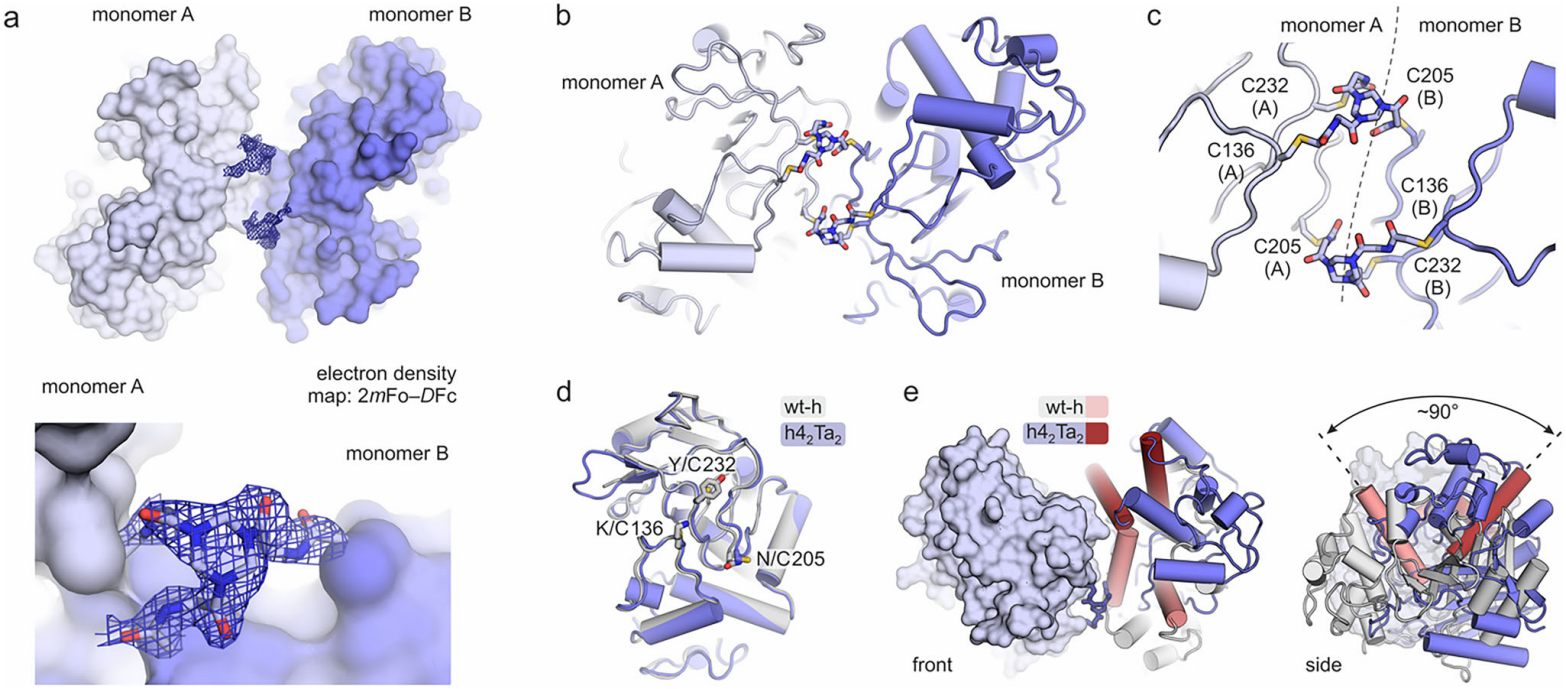
Crystal structure of dimeric h4_2_Ta_2_ (PDB ID 9s7k): (a) Top: h4_2_Ta_2_ dimer in surface representation. Bottom: zoom‐in on one of the cross‐links (mesh: electron density map 2*m*Fo–*D*Fc). (b) h4_2_Ta_2_ dimer in cartoon representation with cross‐links shown as sticks. (c) Zoom‐in on the two cross‐links. (d) Overlay of wt‐h with one of the h4_2_Ta_2_ monomers (RMSD = 0.393 Å, cross‐link not shown). (e) Overlay of the crystallographic wt‐h dimer (gray) and h4_2_Ta_2_ (blue). For reference, two analogous helices are indicated in red (light vs. dark for wt‐h and h4_2_Ta_2_, respectively). When overlaying one of the two monomers of wt‐h and h4_2_Ta_2_ the other one appears to be rotated by approximately 90°C.

It is surprising that cross‐linking of variant h4 provides a covalent protein dimer with two fully reacted three‐armed cross‐links (h4_2_Ta_2_). This is noteworthy since the design process considered the enzyme to be present as monomer. To elucidate the structure of this bioconjugated dimer, protein crystallography was pursued starting with commercial crystallization screens in 200 nL sitting drop 96‐well format [38]. Finally, suitable crystals were eventually obtained by optimizing initial hits in 2 µL sitting drops using 48‐well plates. X‐ray diffraction data was collected at the Diamond Light Source (I04 beamline), and two datasets (chi = 0° and 30°) were integrated with XDS, then scaled and merged with a P_scale to 1.77 Å [39, 40, 41]. Molecular replacement with MoRDa (PDB ID 4y7p) [35] provided the solution (space group *P1*, PDB ID 9s7k, Table S2) [42, 43, 44, 45] revealing an asymmetric unit with two enzyme monomers and clearly defined electron density for two cross‐links (Figure 3a). The cross‐links are located close to each other bridging the two monomers (Figure 3b). Cysteines 136 and 232 in monomer A are cross‐linked with C205 of monomer B (Figure 3c) and vice versa. This generates an overall bicyclic topology (Figure S20). Notably, both monomers in h4_2_Ta_2_ overlay closely with each other (RMSD = 0.096 Å, Figure S20) as well as with wt‐h (RMSD = 0.393 Å, Figure 3d).

Taking a more detailed look into the assembly of the dimer h4_2_Ta_2_, we used AlphaFold 3 [46] to predict a homodimeric structure of h4. Interestingly, the modeled dimer overlays well with a dimer assembly found in the crystal structure of wild‐type enzyme wt‐h (Figure S21). Notably, the dimer structure obtained for h4_2_Ta_2_ involves a similar protein interface, however, the orientation of the two monomers to each other is significantly altered (Figure 3e). In fact, when overlaying one of the monomeric units of h4_2_Ta_2_ and wt‐h, the orientation of the second monomer of h4_2_Ta_2_ appears to be rotated by approximately 90° relative to the second monomer of wt‐h (Figure 3e). Importantly, the dimer arrangement predicted for h4 does not allow the double‐cross‐linking topology observed in the crystal structure of h4_2_Ta_2_ since the cysteine residues would not be accessible (Figure S21). It is not clear whether any of these dimer arrangements is present in solution, or if the observed h4_2_Ta_2_ dimer is only formed during cross‐linking. However, the two different dimer arrangements may point toward a relatively weak homodimer interaction which could explain the monomerization in presence of GuHCl observed during cross‐linking toward h4Ta. To assess the multimeric state in solution, we performed SEC of wt‐h and h4_2_Ta_2_ showing a considerable increase in retention volume upon dilution of wt‐h but not in the case of h4_2_Ta_2_ (Figure S22). This indeed suggests a monomer/dimer equilibrium for wt‐h (increased monomer content upon dilution) and a concentration‐independent state for h4_2_Ta_2_ (stable dimer). The presence of a monomer/dimer equilibrium for wt‐h is also supported by the generally lower retention volumes (*V*_r_ = 11.4–12.8 mL) compared to permanent dimer h4_2_Ta_2_ (*V*_r_ = 10.5–10.7 mL, Figure S22).

### 2.4 Double INCYPRO Variant With Superior Performance

**FIGURE 4:**
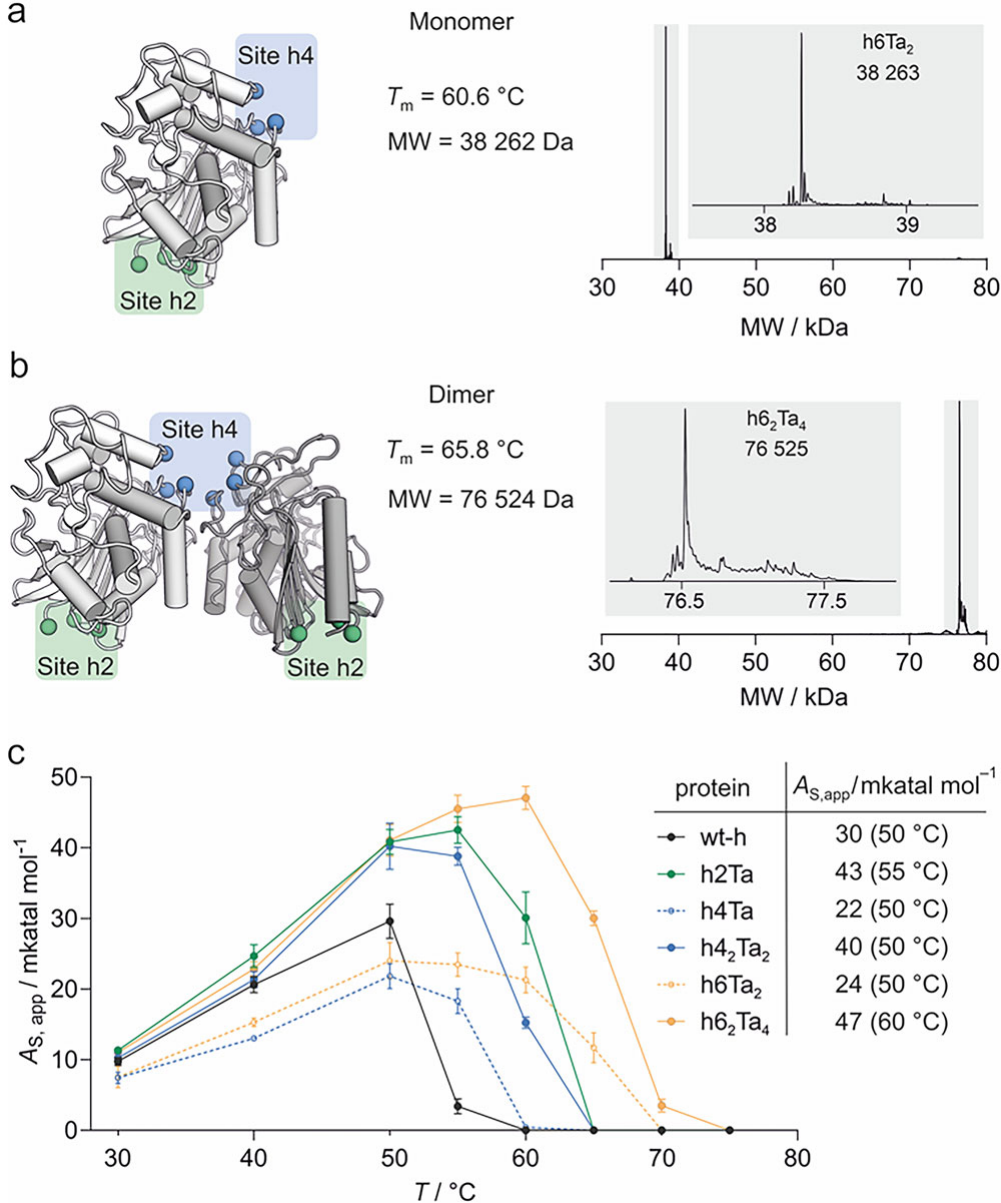
Cross‐linking of INCYPRO double variant: (a) Model of INCYPRO sites in h6Ta_2_ including deconvoluted TOF‐MS (calc. MW = 38262 Da, for spectrum and peak list see Figure S26 and for thermal denaturation curves Figure S28). (b) Model of INCYPRO sites in h6_2_Ta_4_ including deconvoluted TOF‐MS (calc. MW = 76524 Da, for spectra and peak list see Figure S27 and for thermal denaturation curves Figure S28). (c) Temperature‐dependent enzymatic activity (*A*_s,app_, *c*_protein_ = 0.5 µM, based on monomer, *c*_substrate_ = 50 µM, buffer: 100 mM phosphate, pH 8.0, 150 mM NaCl, peptide probe in Figure S19). Table provides maximum activity per protein and corresponding temperature (errors = 1*σ*, *n* ≥ 3, *A*_S,app_‐values in Table S3).

Seeking an enzyme with further increased stability, the structural insights prompted us to combine INCYPRO sites of h2 and h4 which are located on opposite sides of the enzyme and individually provided the most stable and active enzymes. The resulting sextuple cysteine variant h6 was successfully expressed and purified (Figures S23 and S24). In analogy to variant h4, we successfully cross‐linked h6 in its covalent monomer (h6Ta_2_, Figure 4a) and dimer form (h6_2_Ta_4_, Figures 4b and S25). Notably, monomeric h6Ta_2_ exhibits further improved stability (*T*_m_ = 60.6°C, Figure S28) compared to the mono‐cross‐linked precursors h2Ta and h4Ta (Δ*T*_m_ = +2.3 and +8.0°C, Figure 2c). The same trend is seen for the stability of cross‐linked dimer h6_2_Ta_4_ (*T*_m_ = 65.8°C, Figure S28) exceeding its precursors h2Ta and h4_2_Ta_2_ (Δ*T*_m_ = +7.5 and +8.6°C, Figure 2c). This confirms the benefit of combining two INCYPRO sites and renders covalent dimer h6_2_Ta_4_ the most stable enzyme in the entire panel (Δ*T*_m_ = +16°C, relative to wt‐h).

To investigate the effect of increased thermal stability on enzymatic activity, we next performed temperature‐dependent activity measurements with wt‐h and all isolated cross‐linked variants using the earlier described peptide probe and readout (Figure S19). Up to 50°C, increasing temperature promotes enzymatic activity for all enzymes. At 50°C, all cross‐linked variants, except for monomer cross‐linked h4Ta and h6Ta_2_, show higher activity than wt‐h. Notably, the two most stable proteins h2Ta (*T*_m_ = 58.3°C) and h6_2_Ta_4_ (*T*_m_ = 65.8°C) exhibit further increased activity at 55°C. Only h6_2_Ta_4_ exhibits then even further increased activity at 60°C (*A*_S,app_ = 47.1 mkatal mol^−1^) representing the highest enzymatic activity for all tested variants and conditions. The superior performance of dimeric h6_2_Ta_4_ originates from its increased activity compared to wt‐h (at 30°C: *A*_S,app_ = 11.0 vs. 9.8 mkatal mol^−1^) and its higher thermal stability (Δ*T*_m_ = +16°C).

### 2.5 Dimeric h6_2_Ta_4_ is Functional Under Stress

**FIGURE 5:**
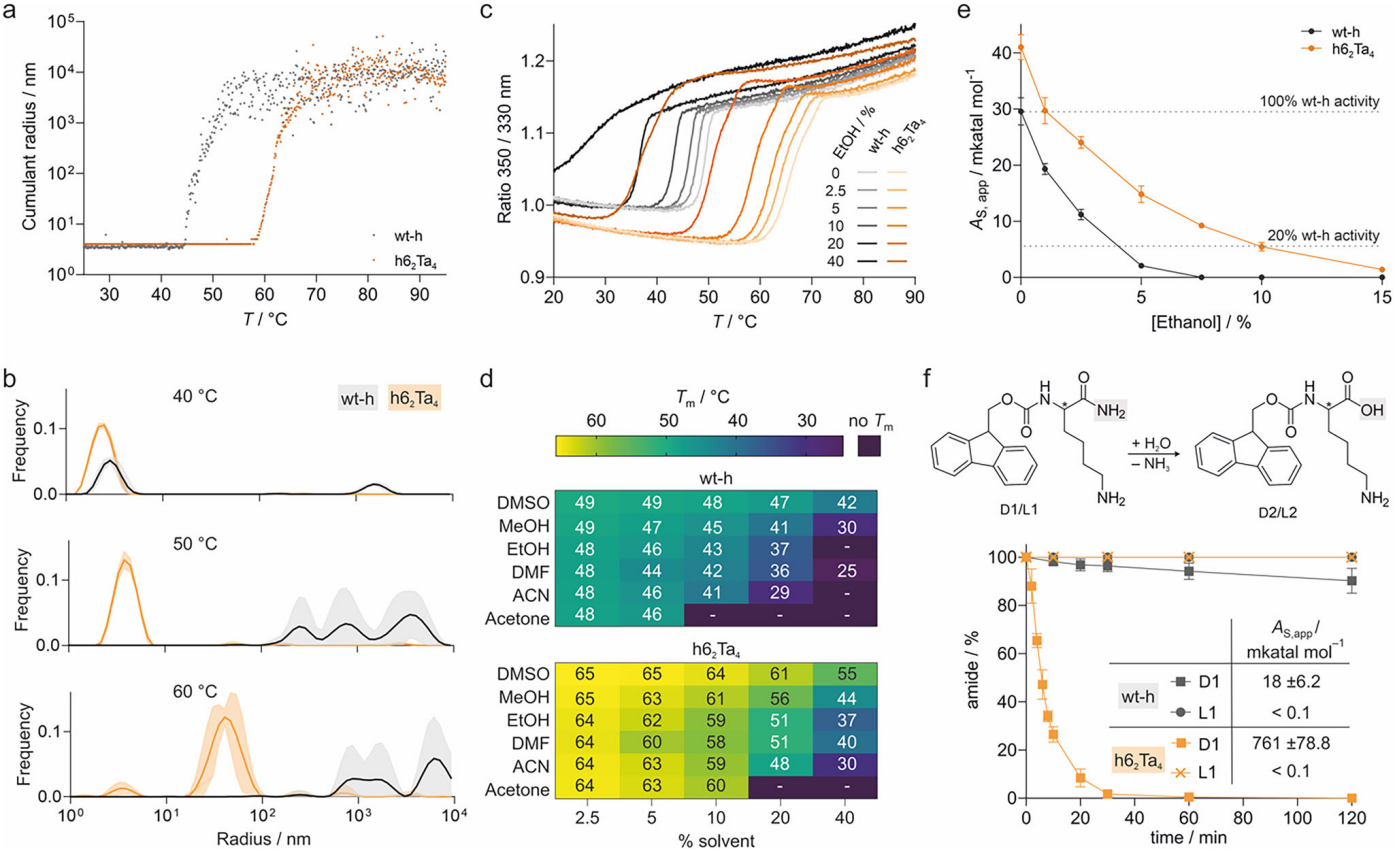
Comparison of wt‐h and h6_2_Ta_4_: (a) Temperature‐dependent cumulant radius of wt‐h (gray) and h6_2_Ta_4_ (orange, buffer: 50 mM HEPES pH 8.0, 50 mM NaCl). (b) DLS‐derived size distribution at different temperatures for wt‐h (gray) and h6_2_Ta_4_ (orange). The errors account for 1*σ* (*n* = 5, buffer: 50 mM HEPES pH 8.0, 50 mM NaCl). For additional temperatures see Figure S29 (errors = 1*σ*, *n* = 5). (c) Thermal denaturation profiles of wt‐h (gray) and h6_2_Ta_4_ (orange), in the presence of varying concentrations of ethanol (0%–40%, *c*_protein_  = 10 µM, based on monomer, buffer: 50 mM HEPES pH 8.0, 50 mM NaCl). (d) Melting temperatures (*T*_m_) in the presence of varying amounts of cosolvent (thermal denaturation curves and *T*_m_‐values in Figures S30 and Table S4). (e) Enzymatic activity (*A*_s,app_) of wt‐h (black) and h6_2_Ta_4_ (orange) in the presence of varying amounts of ethanol at 50°C. For values see Table S5 (errors = 1*σ*, *n* ≥ 3). (f) Hydrolase activity of wt‐h (gray) and h6_2_Ta_4_ (orange) on D‐ and L‐configured substrate (D1 and L1, respectively) using 50 µM Fmoc‐k/K‐NH_2_ (D1/L1) at 50°C in 10% ethanol (UPLC 300 nm readout, *c*_protein_ = 0.1 µM, based on monomer, *c*_substrate_ = 50 µM, buffer: 100 mM phosphate pH 8.0, 150 mM NaCl). For details see Figures S32, S33 and Table S7 (errors = 1*σ*, *n* ≥ 3).

Next, we conducted a detailed characterization of best‐performing cross‐linked enzyme h6_2_Ta_4_ in comparison to wt‐h. To assess their aggregation behavior, temperature‐dependent dynamic light scattering (DLS) measurements were performed (Figure 5a). The onset of aggregation (*T*_on,agg_) for h6_2_Ta_4_ (*T*_on,agg_ = 57.1°C) compared to wt‐h (*T*_on,agg_ = 43.2°C) was considerably delayed (Δ*T*_on,agg_ = +14°C) consistent with their difference in melting temperatures (Δ*T*_m_ = +16°C). A closer inspection of the DLS‐derived size distributions (radius) across different temperatures (Figures 5b and S29) revealed both enzymes to be predominantly monodisperse at 40 °C. At 55°C, h6_2_Ta_4_ maintained a homogeneous population, whereas wt‐h displayed a broad distribution at larger radii indicative of higher‐order aggregates. At 60°C, h6_2_Ta_4_ began to show signs of aggregation, while wt‐h remained in a highly aggregated state. This indicates a reduced aggregation tendency for h6_2_Ta_4_ presumably due to its reinforced protein fold.

To further investigate the stability differences between h6_2_Ta_4_ and wt‐h, we analyzed their thermal melting behavior in the presence of different organic solvents. Thermal denaturation experiments were conducted using DSF as a readout (Figure S30), enabling us to monitor structural changes in the presence of varying concentrations of organic cosolvents (2.5%–40%, Figure 5c, EtOH as an example). While in most cases *T*_m_‐values could be determined, some traces did not allow a reliable assignment of melting temperatures (e.g., wt‐h with 40 % EtOH, Figure 5c). In general, we observed a decline in thermal stability with increasing concentrations of cosolvent (Figure 5d), with aprotic organic solvents, particularly acetone and acetonitrile (ACN), causing the most pronounced destabilization. In all cases where both *T*_m_‐values could be determined, h6_2_Ta_4_ exhibits constantly higher melting temperatures than wt‐h (Δ*T*_m_ = +13 – +19°C, Table S4). These findings further highlight the stabilizing effects of cross‐linking in h6_2_Ta_4_.

Substrate solubility is often an issue for hydrolase applications, which is commonly addressed by the usage of EtOH as cosolvent. For this reason, we assessed the enzymatic activity of h6_2_Ta_4_ and wt‐h at different EtOH concentrations. Based on the high catalytic activity observed for both enzymes at 50°C, we applied it as reaction temperature (Figure 4c). At all EtOH concentrations, we observed higher activity for h6_2_Ta_4_ (Figure 5e). At 5% EtOH, for example, wt‐h exhibits only 2.1% of its activity in absence of cosolvent, whereas h6_2_Ta_4_ maintains 15% of its activity without cosolvent and 21% relative to unstressed wt‐h. At 10 % EtOH and above, wt‐h loses its enzymatic activity while h6_2_Ta_4_ retains activity up to 15% ethanol. Time‐dependent measurements under stressed conditions (10% ethanol at 50°C) revealed a half‐life of 36 h for h6_2_Ta_4_ while wt‐h showed rapid loss of activity with a half‐life of < 5 min (Figure S31).

To further evaluate the catalytic performance, we compared h6_2_Ta_4_ and wt‐h using Fmoc‐protected D‐lysine amide (D1) as substrate which is converted into the corresponding carboxylic acid (D2, Figure 5f). Under unstressed conditions, both enzymes show similar activity (Figure S32 and Table S6). Notably, in the presence of 10% ethanol at 50°C, h6_2_Ta_4_ displayed 42‐fold higher enzymatic activity than wt‐h, confirming above observed stabilizing effects (Table S7). By contrast, no activity was detected for either enzyme when challenged with the non‐cognate substrate based on the natural L‐lysine (L1,) allowing the efficient racemic resolution of D1 and L1 by h6_2_Ta_4_ in the presence of cosolvent at elevated temperature (ee > 99%, Figure S33). We also performed reactions at larger scale, using a racemic mixture of Fmoc‐lysine amide (D1/L1, 1:1, 11 mg, *c* = 2 mM) which was treated with h6_2_Ta_4_ (*c* = 0.1 µM) in buffer (pH 8) supplemented with 10% ethanol (*T* = 50°C). HPLC/MS analysis confirmed the expected 50% conversion (Figure S34) and isolation of product D2 provided 4.8 mg (yield: 44%, theoretical maximum: 50%). Product identity was confirmed by NMR (Figure S34). Taken together these tests highlight the efficiency and stereospecificity of the engineered multicyclic D‐hydrolase h6_2_Ta_4_ and show that INCYPRO‐based stabilization can preserve the intrinsic activity and selectivity of an enzyme while substantially broadening its operational range.

## 3 Conclusions

Hydrolases represent a versatile class of enzymes capable of performing selective transformations with broad potential in industrial and biotechnological applications [47]. Here, we addressed the limitations of a D‐stereospecific hydrolase (DHy) characterized by low thermal stability and high sensitivity to organic cosolvents. By applying the INCYPRO approach, we introduced site‐specific covalent cross‐links that substantially improved the enzyme's resilience. Unexpectedly, we found that the enzyme could be cross‐linked in its dimeric form at a 2:2 protein‐to‐cross‐link ratio (h4_2_Ta_2_), and x‐ray crystallography confirmed the covalently stabilized dimeric structure. Notably, the cross‐linked dimer (h4_2_Ta_2_) exhibited higher thermal stability than the cross‐linked monomer (h4Ta). Building on this finding, we engineered a multicyclic dimeric variant by incorporating two orthogonal cross‐linking sites per monomer, which resulted after cross‐linking in an active dimeric enzyme with superior performance under both thermal and chemical stress. These improvements correlate with a reduced aggregation propensity, consistent with the enhanced structural integrity conferred by macrocyclization. Importantly, stabilization via INCYPRO preserved the intrinsic stereoselectivity of the enzyme while significantly broadening its operational range.

Finding an active DHy in a ‘forced’ dimeric form is interesting, considering that certain proteases have been reported to act as dimers, some even as obligate dimers (e.g. HIV‐1 protease, HTLV‐1 protease, KSHV protease and 3CLpro) [48, 49, 50, 51]. Among structurally characterized proteins with high sequence similarity (≤95%, in Protein Data Bank) [52], we only found D‐stereospecific hydrolases, however, none of them reported to form dimers [33, 35, 53, 54]. Given the observed dimerization of DHy and the high activity of covalent dimers h4_2_Ta_2_ and h6_2_Ta_4_, it is possible that some of these proteases also undergo dimer formation. Furthermore, the ability of h6_2_Ta_4_ to remain active under elevated temperature and in the presence of cosolvent underscores the potential of chemical macrocyclization in particular for the stabilization of protein complexes to unlock the application of otherwise fragile biocatalysts in process‐relevant environments. Taken together, these results establish protein macrocyclization as a powerful strategy for the development of robust enzymes. This is particularly interesting, considering the INCYPRO approach can be combined with sequenced‐based optimization strategies [29], such as FireProt [55, 56], Protein Repair One Stop Shop (PROSS) [57, 58] or ancestral sequence reconstruction [59], which can further add to protein stability and durability [60].

## Conflicts of Interest

S.H., S.N., and T.N.G. are listed as inventors on a patent application related to the cross‐linking of protein complexes. S.N., S.H., and T.N.G. are co‐founders and shareholders of Incircular BV, commercializing the corresponding bioconjugation technology. S.H. and T.N.G. are advisors of Incircular BV.

## Supporting information




**Supporting File 1**: anie71698‐sup‐0001‐SuppMat.Pdf.

## Data Availability

Structural data is available via the PDB and other data via the Supporting Information.
