# Supplementary material for: Multicyclic D‐Stereospecific Hydrolase Dimer With High Sustained Activity
Source: Angew Chem Int Ed Engl. 2026 Mar 20;65(22):e21611. doi: 10.1002/anie.202521611 (PMC13206553; doi:10.1002/anie.202521611)
Supplement: Supplementary file 1 — Supporting File 1: anie71698‐sup‐0001‐SuppMat.Pdf. [file ANIE-65-e21611-s001.pdf]

Supporting Information

Multicyclic D-Stereospecific Hydrolase Dimer with High Sustained Activity

Anissa Haim, Sandra Liebscher *et al.*

Supporting Experimental Procedures

Protein sequences, expression and purification

DHy1 variants were either constructed via Q5 site-directed mutagenesis Kit (New England Biolabs Inc., NEB) using DHy1 wildtype gene in pET-30a(+) as a template. Primers were designed using NEBaseChanger®. After incubation with KLD enzyme mix (NEB, Kinase, Ligase, DpnI) the PCR product was transformed into *E. coli* DHα for amplification. Alternatively, DHy1 variants were purchased (Twist Bioscience) in pET-24d(+). In all cases protein sequences were confirmed via Sanger sequencing. All used expression vectors contain a kanamycin resistance cassette and the IPTG-inducible T7-promoter. For protein biosynthesis purified plasmid DNA was transformed into chemically competent *E. coli* BL21 (DE3). Transformed cells were plated on kanamycin-containing LB agar.

For biosynthesis 50 mL LB medium (containing 50 mg/L kanamycin) were inoculated by a single plasmid-containing *E. coli* BL21 (DE3) colony and incubated overnight at 37°C and 110 rpm. 10 mL of overnight culture was transferred to 500 mL volumes of kanamycin-containing LB medium in a baffled cell culture flask and cultured to an OD_{600nm} of 0.6 prior lac operon induction by addition of IPTG (0.5 mM final concentration). Protein expression was conducted for 3 hours at 30 °C at 110 rpm. *E. coli* cells were harvested by centrifugation (5,200 g, 20 min) and pellet was resuspended in 20 mL lysis buffer (20 mM HEPES, 10 mM NaCl, pH 7.5). Cells were disrupted by sonification (amplitude 50%, 6 x 10 sec) and a final concentration of 10 mM DTT was added. Cell debris were removed by ultracentrifugation (120,000 g, 40 min).

Clarified supernatant was loaded onto a HiTrap SP HP strong cation exchange chromatography column (Cytiva) equilibrated with lysis buffer using an automated Äkta prime chromatography system. Loading was performed using lysis buffer at 2 mL/min, followed by further column washing with the same buffer. Final protein elution used lysis buffer containing Tris (20 mM HEPES, 10 mM NaCl, 200 mM Tris, pH 8.5). Protein-containing elution fractions were analyzed by SDS-Page, pooled and finally loaded onto a size exclusion chromatography column (HiLoad 16/600 Superdex 75 pg, Cytiva) equilibrated with 50 mM HEPES pH 8.0, 50 mM NaCl, 1 mM TCEP. Target protein-containing fractions were identified by SDS-Page, pooled and concentrated using centrifugal filter units (Amicon). Final target proteins were analyzed by LC-MS and frozen for storage at -80°C.

INCYPRO cross-linking

Protein cross-linking was initially carried out by mixing 50 µM of protein (monomer concentration) with 300 µM of TaI_3 (freshly prepared in buffer at a concentration of 3 or 5 mM in 50 mM HEPES, 50 mM, pH 8.0) in a total reaction volume of 75 µL. Prior to cross-linking, the protein stocks were stored in purification buffer (50 mM HEPES pH 8.0, 50 mM NaCl, 0.5–1.0 mM TCEP). The addition of TCEP to the purification buffer was to ensure the presence of reduced cysteine sidechains. The reactions were incubated at room temperature for 2 h. Cross-linking reactions for HPLC/MS analysis were quenched by addition of formic acid (final concentration of 0.08% (v/v)).

For optimization of protein cross-linking to obtain h2Ta, $h4_2Ta_2$, and $h6_2Ta_4$ 50 or 200 µM (see table below) of protein (monomer concentration) was mixed with 150 or 500 µM of TaI_3 (see table) in a total reaction volume of 3–10 mL. The reactions were incubated at room temperature for 2 h. To obtain h4Ta and $h6Ta_2$, 50 µM of protein (monomer concentration) was incubated with 0.75 M GuHCl for 30 minutes, followed by mixing with 300 µM TaI_3 at room temperature for 2 h. For all optimized variants the excess cross-linker was removed by buffer exchange on the Äkta Pure using the 10/300 Superdex 75 or 16/600 Superdex 75 column (Cytiva). Subsequently the cross-linked products were concentrated using an Amicon centrifugal concentrator to at least 100 µM.

Table: Reaction conditions for INCYPRO cross-linking of variants h2, h4 and h6. Protein concentrations refer to the protein monomer.

product	*c*(non-cross-linked)	*c*(TaI_3)	Reaction time
h2Ta	200 µM	500 µM	1 h
h4Ta	50 µM	300 µM	2 h
$h4_2Ta_2$	200 µM	500 µM	2 h
$h6Ta_2$	50 µM	300 µM	2 h
$h6_2Ta_4$	50 µM	150 µM	2 h

SDS PAGE

SDS PAGE samples were prepared by mixing protein with standard Coomassie loading dye and incubated at 95 °C for 5 min. The total loading of protein ranged from 0.05–0.1 nmol. Samples were analyzed by a 4–20% gradient gel (GenScript) for 1 h at 140 V and stained by Instant Blue Coomassie (Abcam).

Protein liquid chromatography coupled with mass spectrometry (LC-MS)

LC-MS Analysis of DHy1 variants and their cross-linked products was performed on an Agilent 6230 ESI-TOF LC/MS (3500 V capillary voltage). Two LC mobile phases were used: A (H_2O, 0.1% v/v formic acid) and B (80% isopropanol, 10% acetonitrile, 10% H_2O, 0.1% formic acid v/v), with injection of 5–10 pmol of protein sample via a 50 x 2.1 mm Agilent AdvanceBio RP mAB reverse-phase C4 column. A 10-min gradient of 0–95% solvent B was applied with a flow rate of 0.3 mL/min. Mass spectra were recorded in the m/z 500–3000 range at a scan rate of 1.0 Hz.

Deconvoluted masses were obtained using the maximum entropy algorithm in Agilent Bioconfirm software with a mass range of 10000–80000 Da and H^+ as the amplifier. Tables for all LC-MS measurements with the calculated (m/z = [MW+Z]/Z) and found values for all predominant peaks are included in the Supplementary Information. The calculated molecular weight of each protein was derived from the Expasy ProtParam molecular weight,[1] excluding the N-terminal methionine residue which is typically cleaved from the expressed protein sequence.[2] Cross-linked monomer and dimer molecular weights were calculated by addition of 378.2 g/mol per cross-link corresponding the mass of fully reacted form of TaI_3, minus the mass of three hydrogens (one per reacted cysteine thiol group).

Thermal stability

Thermal stability was assessed by differential scanning fluorimetry (DSF), measuring the intrinsic fluorescence ratio at λ=350 nm / λ=330 nm. The data was collected on a Prometheus Panta instrument (Nanotemper, T = 25–95 °C, ramp speed = 1 °C/min). Samples were measured at a protein concentration of 50 µM (monomer concentration, 50 mM HEPES pH 8.0, 50 mM NaCl). Errors of the determined T_m-values are generally < 0.5°C and ΔT_ms >1°C can be considered to reflect meaningful differences. Thermal stability in presence of cosolvents DMSO, DMF, acetone, acetonitrile, methanol and ethanol was also assessed with DSF using the Prometheus Panta instrument (Nanotemper, T = 15–75 °C, ramp speed = 1 °C/min). Samples were measured at a protein concentration of 10 µM (monomer concentration, 50 mM HEPES pH 8.0, 50 mM NaCl). The cosolvent concentrations ranged from 0–40% v/v. All capillaries containing acetone, acetonitrile, or methanol were sealed with silicone gum before the measurement to prevent potential sensor damage due to the evaporation of these cosolvents.

Dynamic light scattering (DLS)

Temperature-dependent dynamic light scattering (DLS) measurements were performed using a Prometheus Panta instrument (Nanotemper) at a protein concentration of 50 µM (monomer concentration, 50 mM HEPES pH 8.0, 50 mM NaCl). Measurements were taken at 0.17 °C intervals with a temperature ramp rate of 1 °C/min. The determined autocorrelation function at each given temperature was fitted to obtain a size distribution using Panta Control software. Five size distribution fits within a +0.95 °C range of the temperature were averaged (e.g., data for T = 50 °C represent an average of five measurements between 50.00°C and 50.95°C).

Activity measurements

Enzyme activity was determined using the internally quenched fluorogenic substrate (IQFS) Abz-AAkFAAK(dnp). The IQFS probe bears a 2-aminobenzoic acid (Abz) functionality at its *N*-terminus serving as the fluorescence donor which is combined with 2,4-dinitrophenol (dnp) located at the *C*-terminal end of the substrate. Enzymatic hydrolysis after D-Lys results in an increased fluorescence signal. For fluorescence analysis an excitation wavelength of 320 nm and an emission wavelength of 420 nm were used using a NOVOstar microplate reader (BMG Laboratories, T = 30 °C) and clear-bottom 96-well plates (Corning). Temperature- and solvent-dependent enzyme activity was determined by reversed phase chromatography using a water Acquity UPLC system with diode array detector and ZQ micromass detector. Enzymatic activity was quenched at different time points by the addition of 50% (v/v) acetic acid, and educt/product-ratio was subsequently analyzed using a BEH130 C18 column. Elution was performed with an acetonitrile/water gradient from 0 to 60% (v/v) acetonitrile containing 0.05% formic acid at a flow rate of 0.3 mL/min. Signal quantification was performed at λ = 360 nm. All measurements were performed in 100 mM phosphate buffer pH 8.0, 150 mM NaCl. Buffer and substrate were incubated for 5 min under specified conditions before addition of the respective enzyme. Each data point represents the average of at least three independent measurements.

Size exclusion measurements

Monomeric and dimeric states of the enzyme were analyzed by size-exclusion chromatography (SEC) using an ÄKTA Pure system (Cytiva) equipped with a Superdex 75 Increase 10/300 GL column. The column was equilibrated with 50 mM HEPES pH 8.0, 50 mM NaCl at 4 °C. 200 µL of protein samples were injected and eluted at a flow rate of 0.5 mL/min.

Protein crystallography

Initial screens were set up in 96-well sitting drop IQ plates (stplabtech) with 200 nL drops at a 1:1 ratio of protein to reservoir solution, using a Mosquito (stplabtech). Screens were prepared at room temperature and incubated at either room temperature or 4°C. The best initial hit was found in Morpheus G1 at 4°C.[3] This condition was refined and set up in MRC Maxi 48-well plates (SWISSCI) with 2 µL drops at a 1:1 ratio of protein to reservoir solution, using a Mosquito (stplabtech) at room temperature and incubated at 4°C. The refined condition (100 mM MES/Imidazole pH 7.0, 13 % (v/v) PEG-20000, 26 % (w/v) PEG550-MME, 0.1 M Morpheus Carboxylic Acids) provided crystals that were fished and flash cooled in liquid nitrogen.

Crystals were measured at the I04 beamline at the Diamond Light Source (DLS) at 100 K. Two datasets were collected from one crystal at 0, 30° chi. The data was integrated using XDS (built 20250714)[4] and anisotropically truncated with STARANSIO, using aP_scale.[5] The $h4_2Ta_2$ protein-cross-linker complex was solved on the CCP4 cloud[6] by molecular replacement using MoRDa[7] (PDB ID 4YP7) Iterative rounds of model building was done in COOT[8], and Refmacat.[9] Collection and refinement statistics are listed in Supporting Table S2.

Enzymatic resolution of racemates

For Fmoc-D-Lys-NH_2 and Fmoc-L-Lys-NH_2 a stock solution of 40 mM was prepared using water as solvent. Concentration was adjusted using Fmoc as optical probe (ε = 7500 M^{-1} cm^{-1}). Enzymatic hydrolysis of the amide results in formation of the corresponding acid. Both species can be separated and quantified via UPLC. For this purpose, enzymatic activity was quenched at different time points by the addition of 50% (v/v) acetic acid and educt/product-ratio was subsequently analyzed using a BEH130 C8 column. Elution was performed with an acetonitrile/water gradient from 10 to 80% (v/v) acetonitrile containing 0.05% formic acid at a flow rate of 0.3 mL/min. Peak quantification was performed at λ = 300 nm. To simulate a racemic mixture Fmoc-D-Lys-NH_2 and Fmoc-L-Lys-NH_2 were mixed in equal amounts. Final substrate concentration was 50 µM and enzyme concentration 100 nM. All enzymatic reactions were performed in 100 mM phosphate buffer, 150 mM NaCl, pH 8.0. Buffer and substrate were incubated for 5 min for the specified conditions before addition of the respective enzyme. Each data point represents the average of at least three independent measurements.

For enzymatic resolution of racemates in preparative scale Fmoc-D-Lys-NH_2 and Fmoc-L-Lys-NH_2 were mixed in equal amounts at a final concentration of 2 mM, which is the limit of solubility under these conditions (pH 8.0, 100 mM phosphate buffer pH 8.0, 150 mM NaCl, 10% ethanol, without ethanol

solubility is limited to approximately 1.2 mM). The reaction mixture was incubated with 0.1 µM $h6_2Ta_4$ by stirring in a final volume of 15 mL in a pointed flask placed in a 50°C water bath. Product formation was determined by analyzing 0.5 µL via UPLC-MS at selected time points. After 40 min incubation Fmoc-D-Lys-OH and Fmoc-L-Lys-NH_2 were separated via preparative HPLC using a XSelect® Peptide CSH C18, 10 mm x 250 mm. Elution was performed in 20 min with an acetonitrile/water gradient from 5 to 90% (v/v) acetonitrile containing 0.1% trifluoracetic acid at a flow rate of 3 mL/min. Isolated Fmoc-D-Lys-OH product was finally analyzed via LC-MS and NMR. ^1H NMR spectrum was recorded at 400 MHz in MeOD. Chemical shifts (δ) are reported in ppm relative to internal TMS and coupling constants (J) are given in Hz.

Enzyme stability measurements

For determination of half-life, 10 µM enzyme was incubated in 100 mM phosphate buffer pH 8.0, 150 mM NaCl, 10% ethanol at 50°C in Protein LoBind® reaction vessels (Eppendorf). After selected time points, aliquots were taken for determining enzyme activity using the internally quenched fluorogenic substrate Abz-AAkFAAK(dnp). For fluorescence analysis, an excitation wavelength of 320 nm and an emission wavelength of 420 nm were used using a NOVOstar microplate reader (BMG Laboratories) and clear-bottom 96-well non-binding plates (Greiner). Each well contains 50 µM substrate, 100 mM phosphate buffer pH 8.0, 150 mM NaCl and 0.5 µM enzyme. Enzyme activity was measured at 30°C in a final volume of 150 µL.

Supporting Figures

wt-h

MSSLQTSTQSDRTSVKKAIRDELQLGYPGILAQISKGGKTWSYTAGIADLRTKKPMKADFRFRIGSVT
KTFIATVLLQLSGENRLNLDDSIEKWLPGVIQGNGYDGNQITIRQILNHTSGIADYINSKDFDIMDTK
KSYTAEEFVKMGISLPPDFAPGKGWSYSNTGYVLLGILIEKVTGNSYAEEVENRIIEPLDLSNTFLPG
NSSVIPGTKHARGYLQLDGASELKDVTYINPGSSDGDMISTADDLNKFFSYLLGGKLLKEQQLKQMLT
TVPTNREGTGYGLGILEIKLPNGVSVWGHRGGVLGFSTFAGGTLGGKHTLAINSNSFNINNPESFKNV
LIAEFSK

h1 (K36C/V296C/L316C)

MSSLQTSTQSDRTSVKKAIRDELQLGYPGILAQIS**C**GGKTWSYTAGIADLRTKKPMKADFRFRIGSVT
KTFIATVLLQLSGENRLNLDDSIEKWLPGVIQGNGYDGNQITIRQILNHTSGIADYINSKDFDIMDTK
KSYTAEEFVKMGISLPPDFAPGKGWSYSNTGYVLLGILIEKVTGNSYAEEVENRIIEPLDLSNTFLPG
NSSVIPGTKHARGYLQLDGASELKDVTYINPGSSDGDMISTADDLNKFFSYLLGGKLLKEQQLKQMLT
TVPTNREGTGYGLGILEIKLPNG**C**SVWGHRGGVLGFSTFAGGT**C**GGKHTLAINSNSFNINNPESFKNV
LIAEFSK

h2 (D11C/K36C/K347C)

MSSLQTSTQS**C**RTSVKKAIRDELQLGYPGILAQIS**C**GGKTWSYTAGIADLRTKKPMKADFRFRIGSVT
KTFIATVLLQLSGENRLNLDDSIEKWLPGVIQGNGYDGNQITIRQILNHTSGIADYINSKDFDIMDTK
KSYTAEEFVKMGISLPPDFAPGKGWSYSNTGYVLLGILIEKVTGNSYAEEVENRIIEPLDLSNTFLPG
NSSVIPGTKHARGYLQLDGASELKDVTYINPGSSDGDMISTADDLNKFFSYLLGGKLLKEQQLKQMLT
TVPTNREGTGYGLGILEIKLPNGVSVWGHRGGVLGFSTFAGGTLGGKHTLAINSNSFNINNPESFKNV
LIAEFS**C**

h3 (K57C/K213C/D247C)

MSSLQTSTQSDRTSVKKAIRDELQLGYPGILAQISKGGKTWSYTAGIADLRTKKPM**C**ADFRFRIGSVT
KTFIATVLLQLSGENRLNLDDSIEKWLPGVIQGNGYDGNQITIRQILNHTSGIADYINSKDFDIMDTK
KSYTAEEFVKMGISLPPDFAPGKGWSYSNTGYVLLGILIEKVTGNSYAEEVENRIIEPLDLSNTFLPG
NSSVIPGT**C**HARGYLQLDGASELKDVTYINPGSSDGDMISTA**C**DLNKFFSYLLGGKLLKEQQLKQMLT
TVPTNREGTGYGLGILEIKLPNGVSVWGHRGGVLGFSTFAGGTLGGKHTLAINSNSFNINNPESFKNV
LIAEFSK

h4 (K136C/N205C/Y232C)

MSSLQTSTQSDRTSVKKAIRDELQLGYPGILAQISKGGKTWSYTAGIADLRTKKPMKADFRFRIGSVT
KTFIATVLLQLSGENRLNLDDSIEKWLPGVIQGNGYDGNQITIRQILNHTSGIADYINSKDFDIMDT**C**
KSYTAEEFVKMGISLPPDFAPGKGWSYSNTGYVLLGILIEKVTGNSYAEEVENRIIEPLDLSNTFLPG
CSSVIPGTKHARGYLQLDGASELKDVT**C**INPGSSDGDMISTADDLNKFFSYLLGGKLLKEQQLKQMLT
TVPTNREGTGYGLGILEIKLPNGVSVWGHRGGVLGFSTFAGGTLGGKHTLAINSNSFNINNPESFKNV
LIAEFSK

h5 (T140C/S182C/N205C)

MSSLQTSTQSDRTSVKKAIRDELQLGYPGILAQISKGGKTWSYTAGIADLRTKKPMKADFRFRIGSVT
KTFIATVLLQLSGENRLNLDDSIEKWLPGVIQGNGYDGNQITIRQILNHTSGIADYINSKDFDIMDTK
KSY**C**AEEFVKMGISLPPDFAPGKGWSYSNTGYVLLGILIEKVTGN**C**YAEEVENRIIEPLDLSNTFLPG
CSSVIPGTKHARGYLQLDGASELKDVTYINPGSSDGDMISTADDLNKFFSYLLGGKLLKEQQLKQMLT
TVPTNREGTGYGLGILEIKLPNGVSVWGHRGGVLGFSTFAGGTLGGKHTLAINSNSFNINNPESFKNV
LIAEFSK

Supporting Figure S1: Sequences of DHy1 wt and variants.

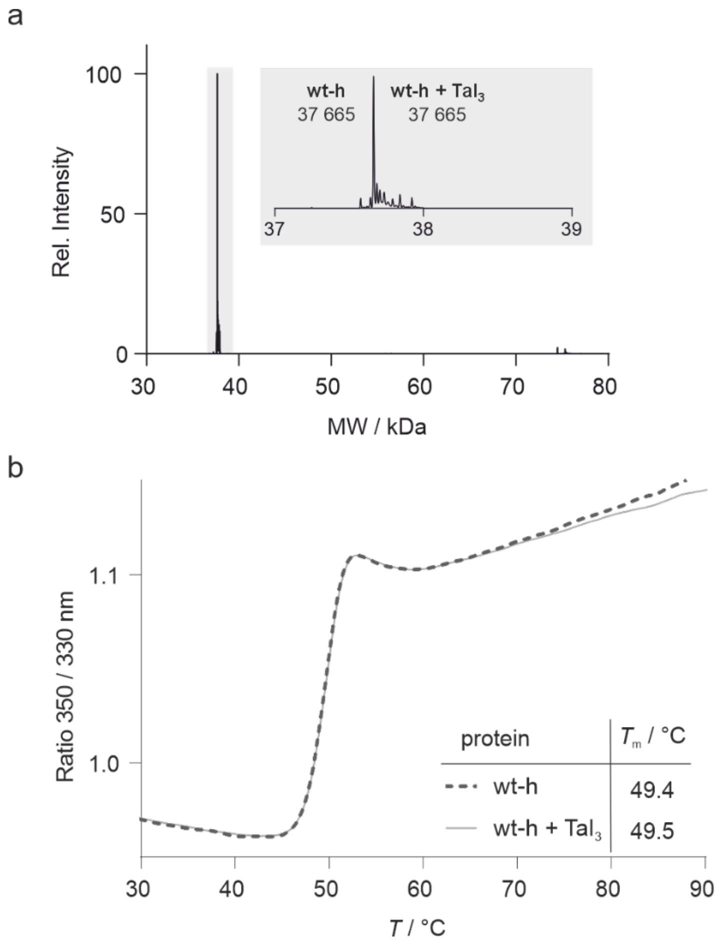

Supporting Figure S2: Characterization of wt-h. a) Deconvoluted TOF-MS spectra of wt-h (calc. MW = 37664 Da, light grey) before and after TaI_3-treatment (dark grey). Data collected on Agilent LC/MSD XT ESI-Quadrupole LC-MS. b) DSF thermal denaturation curves of wt-h before and after TaI_3-treatment. Measured at a 50 µM monomer concentration (in 50 mM HEPES pH 8.0, 50 mM NaCl). For ESI-MS spectrum and peak list, see Supporting Figure S8.

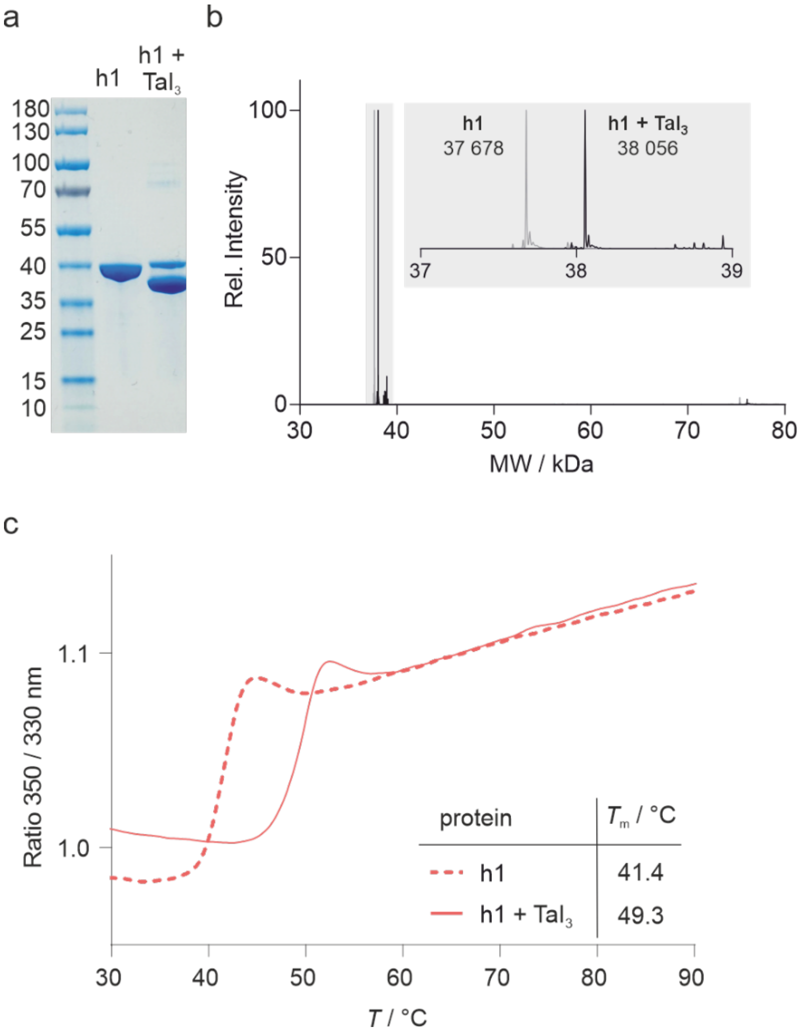

Supporting Figure S3: Characterization of variant h1. a) SDS PAGE of variant h1 and h1 + TaI_3. b) ESI-MS spectrum of h1 (calc. MW = 37675 Da, light grey) before and after TaI_3-treatment (h1Ta: calc. MW = 38053 Da, black). Data collected on Agilent LC/MSD XT ESI-Quadrupole LC-MS. c) DSF thermal denaturation curves of h1 and h1 + TaI_3. Measured at a 50 µM monomer concentration (in 50 mM HEPES pH 8.0, 50 mM NaCl). For ESI-MS spectrum and peak list, see Supporting Figure S9.

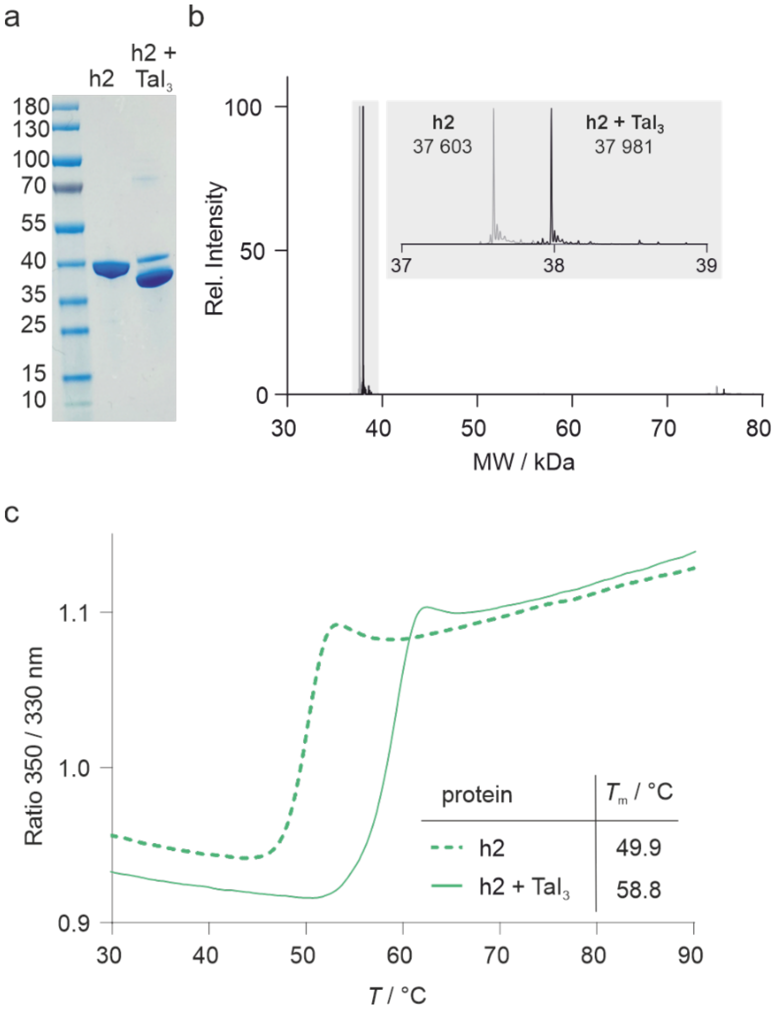

Supporting Figure S4: Characterization of variant h2. a) SDS PAGE of variant h2 and h2 + TaI_3. b) ESI-MS spectrum of h2 (calc. MW = 37602 Da, light grey) before and after TaI_3-treatment (h2Ta: calc. MW = 37980 Da, black). Data collected on Agilent LC/MSD XT ESI-Quadrupole LC-MS. c) DSF thermal denaturation curves of h2 and h2 + TaI_3. Measured at a 50 µM monomer concentration (in 50 mM HEPES pH 8.0, 50 mM NaCl). For ESI-MS spectrum and peak list, see Supporting Figure S10.

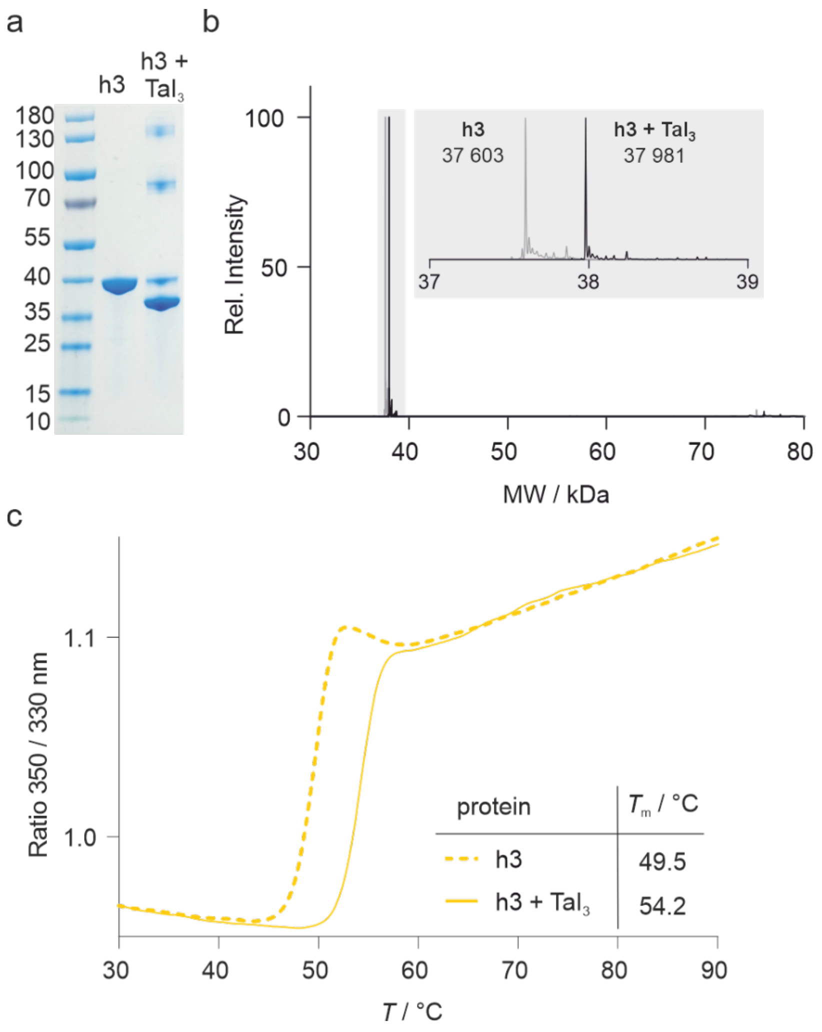

Supporting Figure S5: Characterization of variant h3. a) SDS PAGE of variant h3 and h3 + TaI_3. b) ESI-MS spectrum of h3 (calc. MW = 37602 Da, light grey) before and after TaI_3-treatment (h3Ta: calc. MW = 37980 Da, black). Data collected on Agilent LC/MSD XT ESI-Quadrupole LC-MS. c) DSF thermal denaturation curves of h3 and h3 + TaI_3. Measured at a 50 µM monomer concentration (in 50 mM HEPES pH 8.0, 50 mM NaCl). For ESI-MS spectrum and peak list, see Supporting Figure S11.

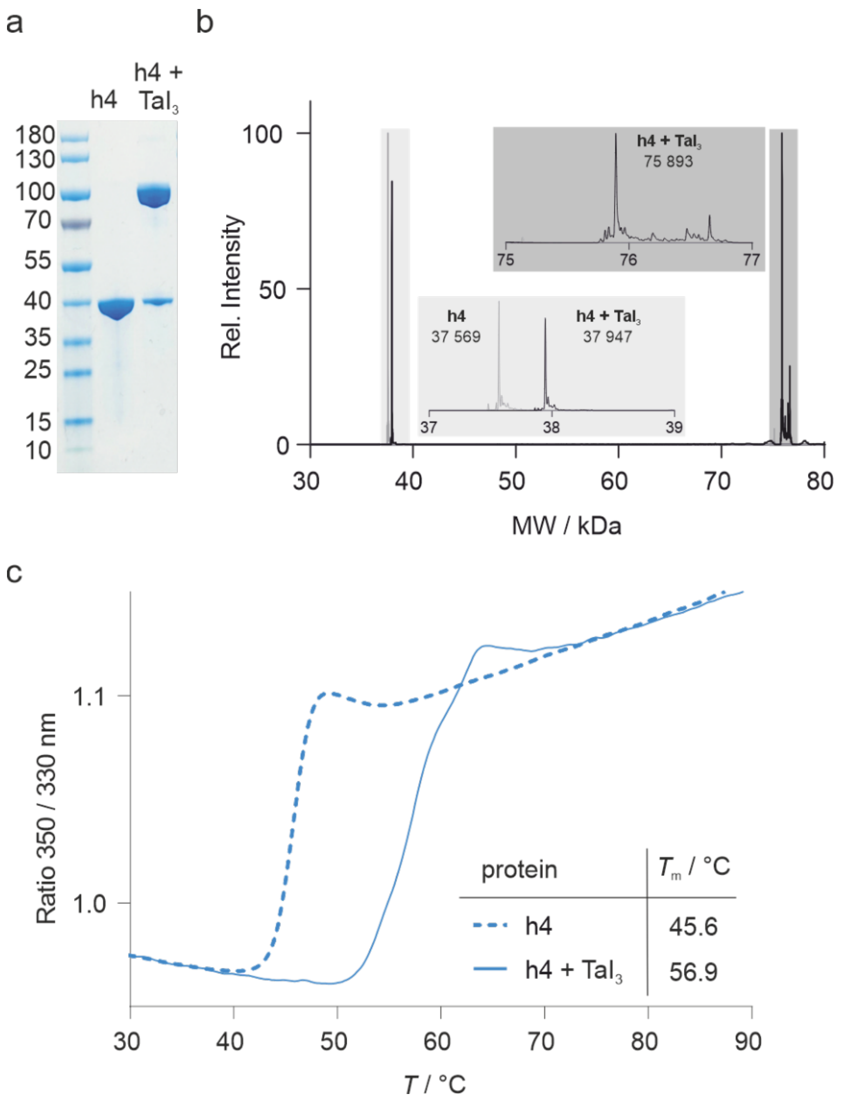

Supporting Figure S6: Characterization of variant h4. a) SDS PAGE of variant h4 and h4 + TaI_3. b) ESI-MS spectrum of h4 (calc. MW = 37568 Da, light grey) before and after TaI_3-treatment (h4Ta: calc. MW = 37946 Da, black). Data collected on Agilent LC/MSD XT ESI-Quadrupole LC-MS. c) DSF thermal denaturation curves of h4 and h4 + TaI_3. Measured at a 50 µM monomer concentration (in 50 mM HEPES pH 8.0, 50 mM NaCl). For ESI-MS spectrum and peak list, see Supporting Figure S12.

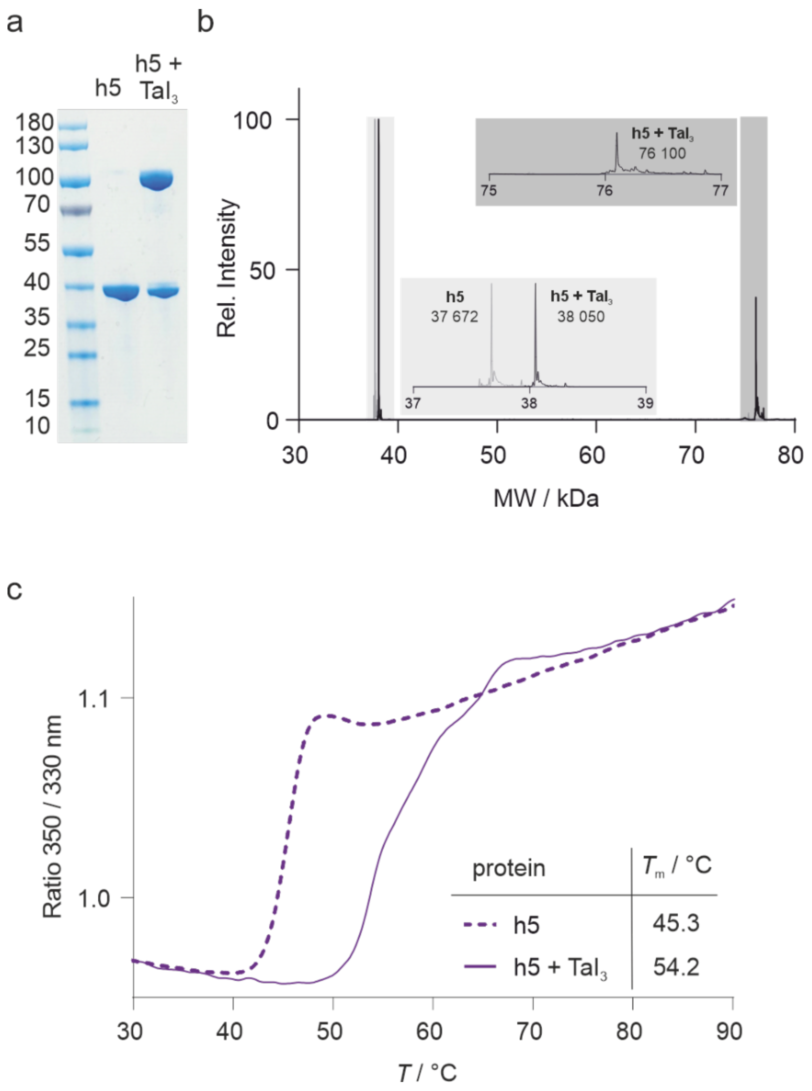

Supporting Figure S7: Characterization of variant h5. a) SDS PAGE of variant h5 and h5 + TaI_3. b) ESI-MS spectrum of h5 (calc. MW = 37671 Da, light grey) and before and after TaI_3-treatment (h5Ta: calc. MW = 38049 Da, black). For variant h5 a mass of a fully cross-linker dimer ($h5_2Ta_2$) was observed (calc. MW = 76098 Da, dark grey). Data collected on Agilent LC/MSD XT ESI-Quadrupole LC-MS. c) DSF thermal denaturation curves of h5 and h5 + TaI_3. Measured at a 50 µM monomer concentration (in 50 mM HEPES pH 8.0, 50 mM NaCl). For ESI-MS spectrum and peak list, see Supporting Figure S13.

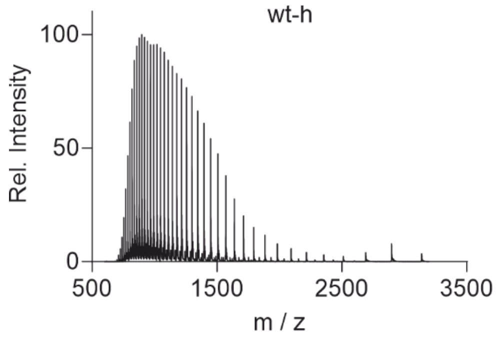

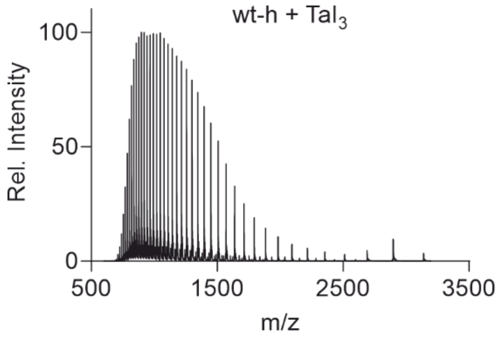

wt-h
calc.: 37663.7 Da
found: 37664.8 Da

wt-h + TaI_3
calc.: 37663.7 Da
found: 37664.8 Da

wt-h		
**Charge**	**m/z found $[M+H]^+$**	**m/z calc.**
50	754.3	754.3
49	769.7	769.7
48	785.7	785.7
47	802.4	802.4
46	819.8	819.8
45	838.0	838.0
44	857.0	857.0
43	876.9	876.9
42	897.8	897.8
41	919.7	919.6
40	942.6	942.6
39	966.8	966.7
38	992.2	992.2
37	1019.0	1018.9
36	1047.3	1047.2
35	1077.1	1077.1
34	1108.8	1108.8
33	1142.4	1142.3
32	1178.0	1178.0
31	1216.0	1216.0
30	1256.5	1256.5
29	1299.8	1299.8
28	1346.2	1346.1
27	1396.0	1396.0
26	1449.6	1449.6
25	1507.6	1507.6

wt-h + TaI_3		
**Charge**	**m/z found $[M+H]^+$**	**m/z calc.**
50	754.3	754.3
49	769.7	769.7
48	785.7	785.7
47	802.4	802.4
46	819.8	819.8
45	838.0	838.0
44	857.0	857.0
43	876.9	876.9
42	897.8	897.8
41	919.7	919.6
40	942.6	942.6
39	966.8	966.7
38	992.2	992.2
37	1019.0	1018.9
36	1047.3	1047.2
35	1077.1	1077.1
34	1108.8	1108.8
33	1142.4	1142.3
32	1178.0	1178.0
31	1216.0	1216.0
30	1256.5	1256.5
29	1299.8	1299.8
28	1346.2	1346.1
27	1396.0	1396.0
26	1449.6	1449.6
25	1507.6	1507.6

Supporting Figure S8: ESI-MS spectrum and peak list for wt-h before and after TaI_3 treatment. Data collected on Agilent LC/MSD XT ESI-Quadrupole LC-MS.

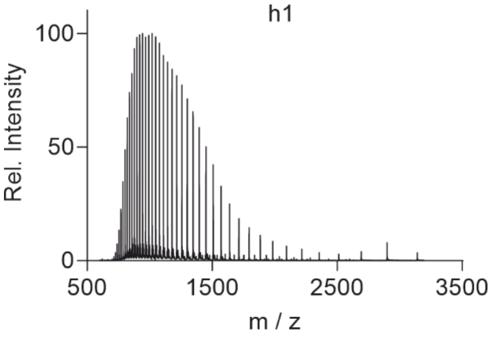

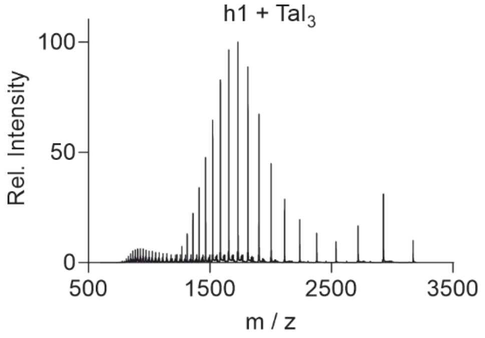

h1 (K36C/V296C/L316C)
calc.: 37675.0 Da
found: 37677.9 Da

h1 (K36C/V296C/L316C) + TaI$_3$
calc.: 38053.1 Da
found: 38055.7 Da

**h1**		
**Charge**	**m/z found [M+H]$^+$**	**m/z calc.**
50	754.6	1508.1
49	770.0	1450.2
48	786.0	1396.5
47	802.7	1346.7
46	820.1	1300.2
45	838.3	1256.9
44	857.3	1216.4
43	877.2	1178.4
42	898.1	1142.8
41	920.0	1109.2
40	943.0	1077.5
39	967.1	1047.6
38	992.5	1019.3
37	1019.3	992.5
36	1047.6	967.1
35	1077.5	943.0
34	1109.2	920.0
33	1142.8	898.1
32	1178.4	877.2
31	1216.4	857.3
30	1256.9	838.3
29	1300.2	820.1
28	1346.6	802.7
27	1396.5	786.0
26	1450.1	769.9
25	1508.1	754.6

**h1 + TaI$_3$**		
**Charge**	**m/z found [M+H]$^+$**	**m/z calc.**
28	1360.1	1360.0
27	1410.5	1410.4
26	1464.7	1464.6
25	1523.2	1523.1
24	1586.7	1586.6
23	1655.6	1655.5
22	1730.8	1730.7
21	1813.2	1813.1
20	1903.8	1903.7
19	2003.9	2003.8
18	2115.2	2115.1

Supporting Figure S9: ESI-MS spectrum and peak list for h1 before and after TaI$_3$ treatment. Data collected on Agilent LC/MSD XT ESI-Quadrupole LC-MS.

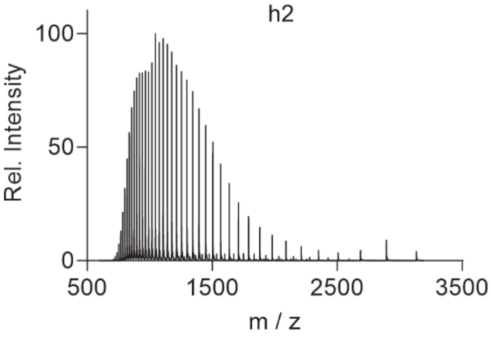

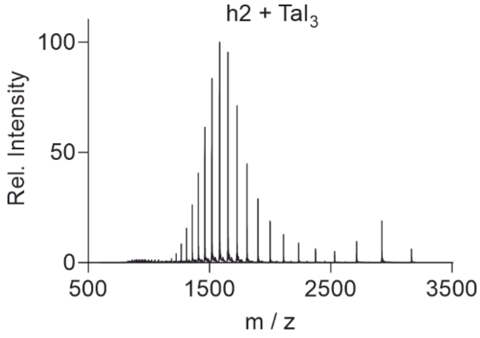

h2 (D11C/K36C/K347C)
calc.: 37601.6 Da
found: 37602.8 Da

h2 (D11C/K36C/K347C) + TaI_3
calc.: 37979.8 Da
found: 37980.7 Da

h2		
**Charge**	**m/z found $[M+H]^+$**	**m/z calc.**
50	1505.1	1505.1
49	1447.3	1447.2
48	1393.7	1393.7
47	1343.9	1343.9
46	1297.6	1297.6
45	1254.4	1254.4
44	1214.0	1214.0
43	1176.1	1176.1
42	1140.5	1140.5
41	1107.0	1106.9
40	1075.4	1075.3
39	1045.5	1045.5
38	1017.3	1017.3
37	990.6	990.5
36	965.2	965.2
35	941.1	941.0
34	918.2	918.1
33	896.3	896.3
32	875.5	875.5
31	855.6	855.6
30	836.6	836.6
29	818.5	818.4
28	801.1	801.0
27	784.4	784.4
26	768.4	768.4
25	753.1	753.0

h2 + TaI_3		
**Charge**	**m/z found $[M+H]^+$**	**m/z calc.**
28	1357.5	1357.4
27	1407.7	1407.7
26	1461.8	1461.8
25	1520.2	1520.2
24	1583.5	1583.5
23	1652.3	1652.3
22	1727.4	1727.4
21	1809.6	1809.6
20	1900.0	1900.0
19	2000.0	1999.9
18	2111.0	2111.0
17	2235.1	2235.1

Supporting Figure S10: ESI-MS spectrum and peak list for h2 before and after TaI_3 treatment. Data collected on Agilent LC/MSD XT ESI-Quadrupole LC-MS.

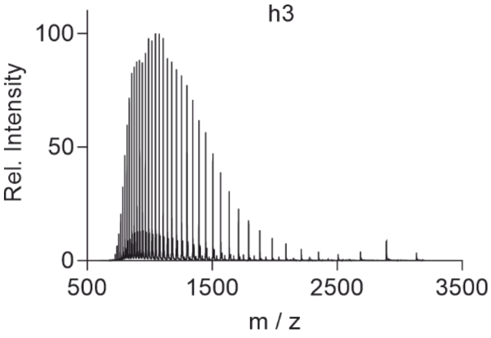

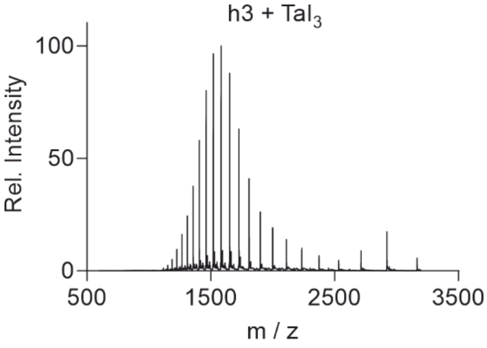

h3 (K57C/K213C/D247C)
calc.: 37601.6 Da
found: 37602.8 Da

h3		
**Charge**	**m/z found [M+H]⁺**	**m/z calc.**
50	753.1	753.0
49	768.4	768.4
48	784.4	784.4
47	801.1	801.0
46	818.5	818.4
45	836.6	836.6
44	855.6	855.6
43	875.5	875.5
42	896.3	896.3
41	918.2	918.1
40	941.1	941.0
39	965.2	965.2
38	990.6	990.5
37	1017.3	1017.3
36	1045.5	1045.5
35	1075.4	1075.3
34	1107.0	1106.9
33	1140.5	1140.5
32	1176.1	1176.1
31	1214.0	1214.0
30	1254.4	1254.4
29	1297.6	1297.6
28	1344.0	1343.9
27	1393.7	1393.7
26	1447.3	1447.2
25	1505.1	1505.1

h3 (K57C/K213C/D247C) + TaI_3
calc.: 37979.8 Da
found: 37980.7 Da

h3 + TaI_3		
**Charge**	**m/z found [M+H]⁺**	**m/z calc.**
29	1310.7	1310.7
28	1357.5	1357.4
27	1407.7	1407.7
26	1461.8	1461.8
25	1520.2	1520.2
24	1583.5	1583.5
23	1652.3	1652.3
22	1727.4	1727.4
21	1809.6	1809.6
20	1900.0	1900.0
19	2000.0	1999.9
18	2111.0	2111.0
17	2235.1	2235.1

Supporting Figure S11: ESI-MS spectrum and peak list for h3 before and after TaI_3 treatment. Data collected on Agilent LC/MSD XT ESI-Quadrupole LC-MS.

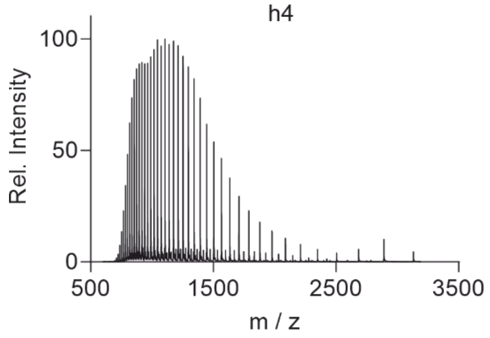

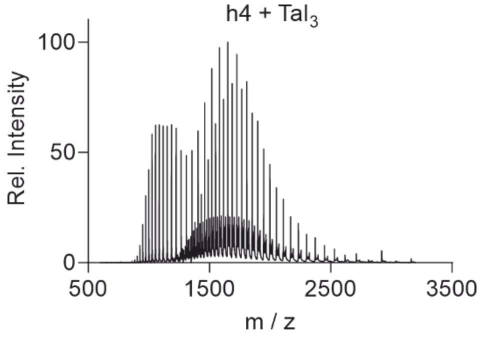

h4 (K136C/N205C/Y232C)
calc.: 37567.6 Da
found: 37568.8 Da

h4 (K136C/N205C/Y232C) + TaI_3
calc.: 37945.8 / 75891.6 Da (monomer/dimer)
found: 37946.8 / 75893.1 Da (monomer/dimer)

h4

Charge	m/z found $[M+H]^+$	m/z calc.
50	752.4	752.4
49	767.7	767.7
48	783.7	783.7
47	800.4	800.3
46	817.7	817.7
45	835.9	835.8
44	854.9	854.8
43	874.7	874.7
42	895.5	895.5
41	917.3	917.3
40	940.2	940.2
39	964.3	964.3
38	989.7	989.6
37	1016.4	1016.3
36	1044.6	1044.6
35	1074.4	1074.4
34	1106.0	1105.9
33	1139.5	1139.4
32	1175.0	1175.0
31	1212.9	1212.9
30	1253.3	1253.3
29	1296.5	1296.4
28	1342.7	1342.7
27	1392.4	1392.4
26	1445.9	1445.9
25	1503.7	1503.7

h4 + TaI_3 monomer

Charge	m/z found $[M+H]^+$	m/z calc.
38	999.6	999.6
37	1026.6	1026.6
36	1055.1	1055.1
35	1085.2	1085.2
34	1117.1	1117.1
33	1150.9	1150.9
32	1186.8	1186.8
31	1225.1	1225.1
30	1265.9	1265.9
29	1309.5	1309.5
28	1356.3	1356.2
27	1406.5	1406.4
26	1460.5	1460.5

h4 + TaI_3 dimer

Charge	m/z found $[M+H]^+$	m/z calc.
48	1582.1	1582.1
47	1615.8	1615.7
46	1650.9	1650.8
45	1687.5	1687.5
44	1725.9	1725.8
43	1766.0	1765.9
42	1808.0	1808.0
41	1852.1	1852.0
40	1898.3	1898.3
39	1947.0	1946.9
38	1998.2	1998.2
37	2052.2	2052.1
36	2109.2	2109.1

Supporting Figure S12: ESI-MS spectrum and peak list for h4 before and after TaI_3 treatment. Data collected on Agilent LC/MSD XT ESI-Quadrupole LC-MS.

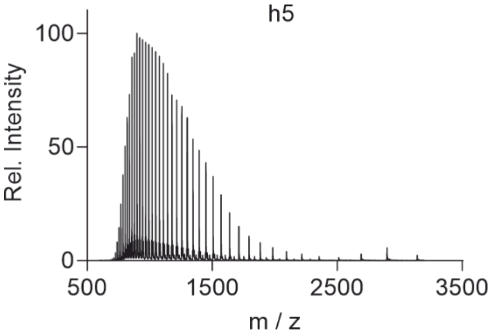

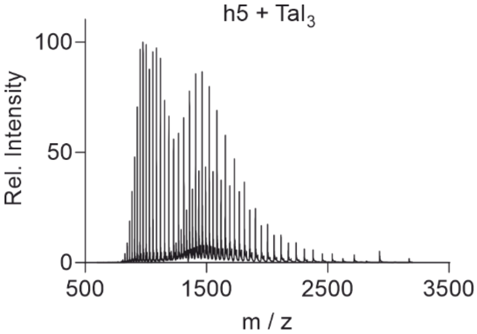

h5 (T140C/S182C/N205C)
calc.: 37670.8 Da
found: 37671.9 Da

h5 (T140C/S182C/N205C) + TaI_3
calc.: 38048.9 / 76097.8 Da (monomer/dimer)
found: 38050.2 / 76099.7 Da (monomer/dimer)

h5

Charge	m/z found $[M+H]^+$	m/z calc.
50	754.5	754.4
49	769.8	769.8
48	785.9	785.8
47	802.6	802.5
46	820.0	819.9
45	838.2	838.1
44	857.2	857.2
43	877.1	877.1
42	898.0	897.9
41	919.8	919.8
40	942.8	942.8
39	967.0	966.9
38	992.4	992.3
37	1019.2	1019.1
36	1047.5	1047.4
35	1077.3	1077.3
34	1109.0	1109.0
33	1142.6	1142.5
32	1178.3	1178.2
31	1216.2	1216.2
30	1256.7	1256.7
29	1300.0	1300.0
28	1346.4	1346.4
27	1396.3	1396.2
26	1449.9	1449.9
25	1507.9	1507.8

h5 + TaI_3 monomer

Charge	m/z found $[M+H]^+$	m/z calc.
38	1002.3	1002.3
37	1029.4	1029.4
36	1058.0	1057.9
35	1088.2	1088.1
34	1120.1	1120.1
33	1154.0	1154.0
32	1190.1	1190.0
31	1228.4	1228.4
30	1269.3	1269.3
29	1313.1	1313.0
28	1359.9	1359.9
27	1410.3	1410.2
26	1464.5	1464.4

h5 + TaI_3 dimer

Charge	m/z found $[M+H]^+$	m/z calc.
48	1586.4	1586.4
47	1620.2	1620.1
46	1655.4	1655.3
45	1692.2	1692.1
44	1730.6	1730.5
43	1770.8	1770.7
42	1812.9	1812.9
41	1857.1	1857.1
40	1903.6	1903.5
39	1952.3	1952.2
38	2003.7	2003.6
37	2057.8	2057.7
36	2114.9	2114.8

Supporting Figure S13: ESI-MS spectrum and peak list for h5 before and after TaI_3 treatment. Data collected on Agilent LC/MSD XT ESI-Quadrupole LC-MS.

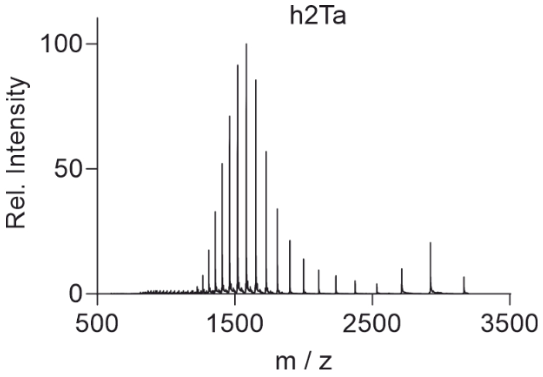

h2Ta
calc.: 37979.8 Da
found: 37980.6 Da

h2Ta		
Charge	m/z found $[M+H]^+$	m/z calc.
28	1357.4	1357.4
27	1407.7	1407.7
26	1461.8	1461.8
25	1520.2	1520.2
24	1583.5	1583.5
23	1652.3	1652.3
22	1727.4	1727.4
21	1809.6	1809.6
20	1900.0	1900.0
19	2000.0	1999.9
18	2111.0	2111.0
17	2235.1	2235.1

Supporting Figure S14: ESI-MS spectrum and peak list for h2Ta. Data collected on Agilent LC/MSD XT ESI-Quadrupole LC-MS.

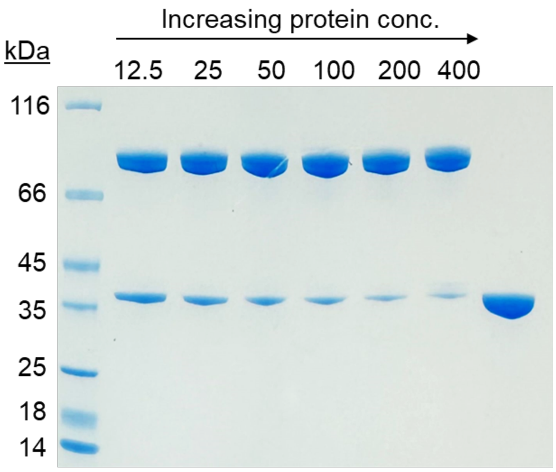

Supporting Figure S15: SDS PAGE gel of optimization of cross-linking reaction of $h4_2Ta_2$. The last well contains variant h4 without addition of TaI_3. Cross-linking with TaI_3 was performed in 50 mM HEPES pH 8.0, 50 mM NaCl, with protein monomer concentrations varying from 12.5–400 µM and TaI_3 concentrations varying from 312.5–700 µM. The reaction time was 1 h. In each well, 0.1 nmol of sample was injected.

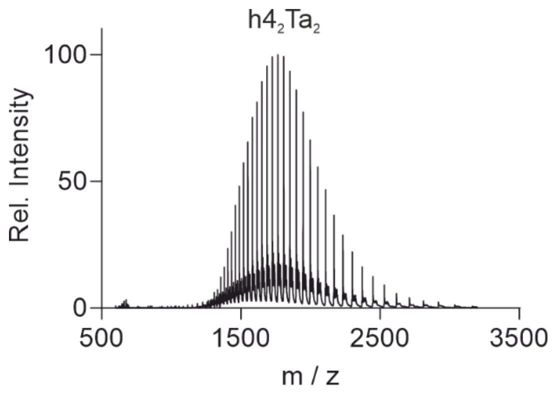

$h4_2Ta_2$
calc.: 75891.6 Da
found: 75892.7 Da

	$h4_2Ta_2$	
Charge	m/z found $[M+H]^+$	m/z calc.
55	1380.9	1380.9
54	1406.5	1406.4
53	1433.0	1432.9
52	1460.5	1460.5
51	1489.2	1489.1
50	1518.9	1518.8
49	1549.9	1549.8
48	1582.2	1582.1
47	1615.8	1615.7
46	1650.9	1650.8
45	1687.6	1687.5
44	1725.9	1725.8
43	1766.0	1765.9
42	1808.0	1808.0
41	1852.1	1852.0
40	1898.4	1898.3
39	1947.0	1946.9
38	1998.2	1998.2
37	2052.2	2052.1
36	2109.2	2109.1
35	2169.4	2169.3
34	2233.2	2233.1
33	2300.8	2300.8
32	2372.7	2372.6

Supporting Figure S16: ESI-MS spectrum and peak list for $h4_2Ta_2$. Data collected on Agilent LC/MSD XT ESI-Quadrupole LC-MS.

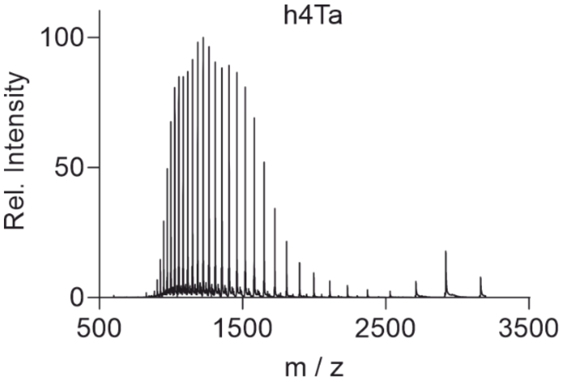

h4Ta
calcd: 37945.8 Da
found: 37946.8 Da

Charge	h4Ta<br>m/z found [M+H]$^+$	m/z calc.
41	926.5	926.5
40	949.7	949.7
39	974.0	974.0
38	999.6	999.6
37	1026.6	1026.6
36	1055.1	1055.1
35	1085.2	1085.2
34	1117.1	1117.1
33	1150.9	1150.9
32	1186.8	1186.8
31	1225.1	1225.1
30	1265.9	1265.9
29	1309.5	1309.5
28	1356.2	1356.2
27	1406.4	1406.4
26	1460.5	1460.5
25	1518.9	1518.8
24	1582.1	1582.1
23	1650.8	1650.8
22	1725.8	1725.8

Supporting Figure S17: ESI-MS spectrum and peak list for h4Ta. Data collected on Agilent LC/MSD XT ESI-Quadrupole LC-MS.

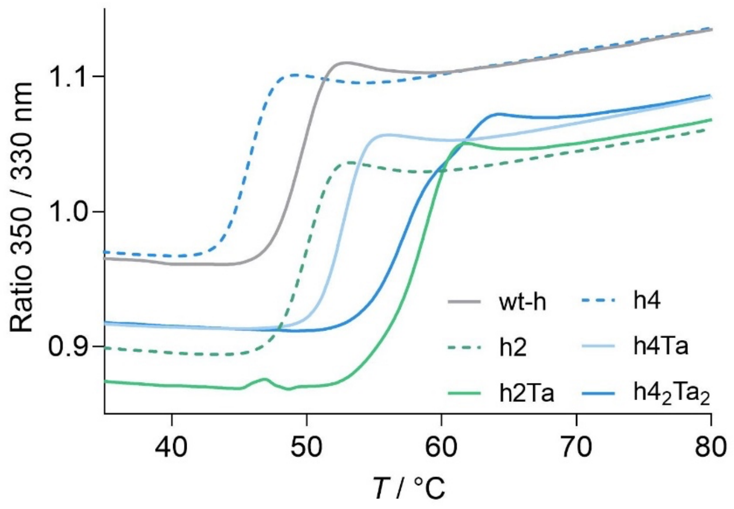

Supporting Figure S18: DSF thermal denaturation curves of wt-h, h2, h2Ta, h4, h4Ta and h4$_2$Ta$_2$ after optimization. Measured at 50 µM monomer concentration (in 50 mM HEPES pH 8.0, 50 mM NaCl).

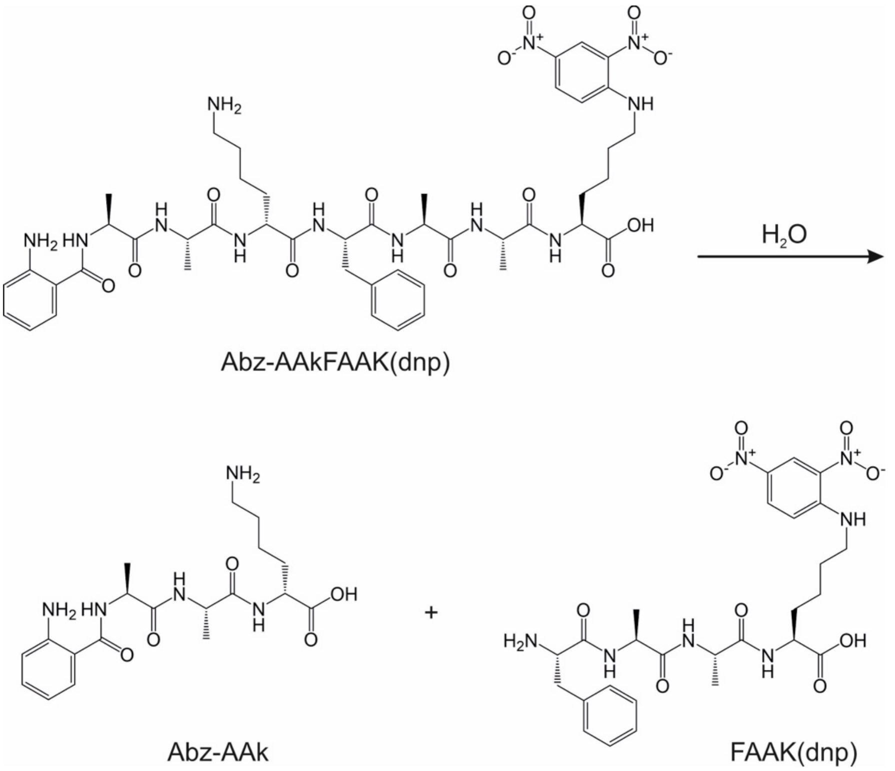

Supporting Figure S19: Chemical structure of the peptide (Abz-AAkFAAK(dnp)) used in the DHy1-hydroslysis measurements (Abz = 2-amino benzoic acid, dnp = 2,4-dinitrophenol).

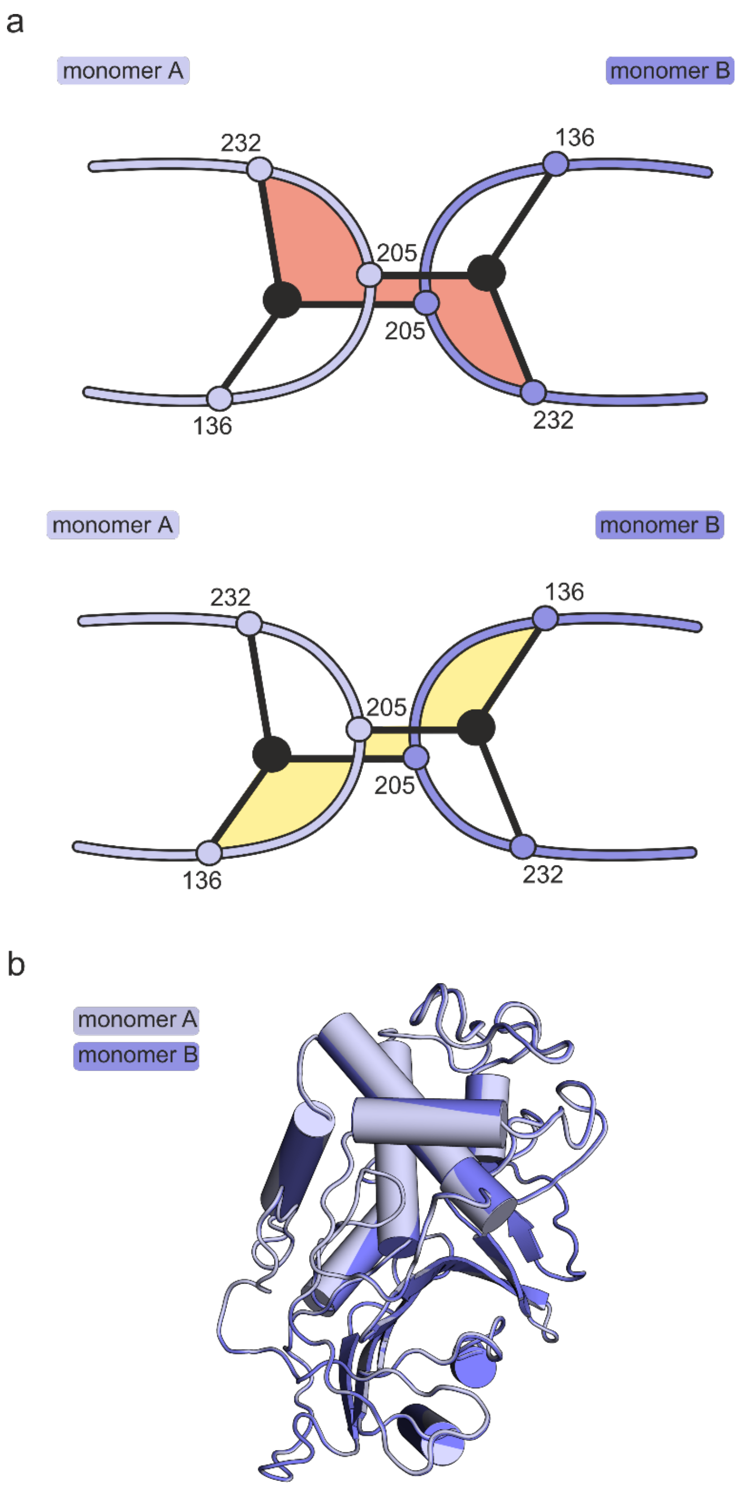

Supporting Figure S20: a) Topology scheme of $h4_2Ta_2$ after cross-linking. The numbers correspond to the cysteine residues in both monomers. The two cycles forming after cross-linking are shown in light red and yellow. b) Overlay of both protomers in $h4_2Ta_2$ in cartoon representation (PDB ID 9s7k).

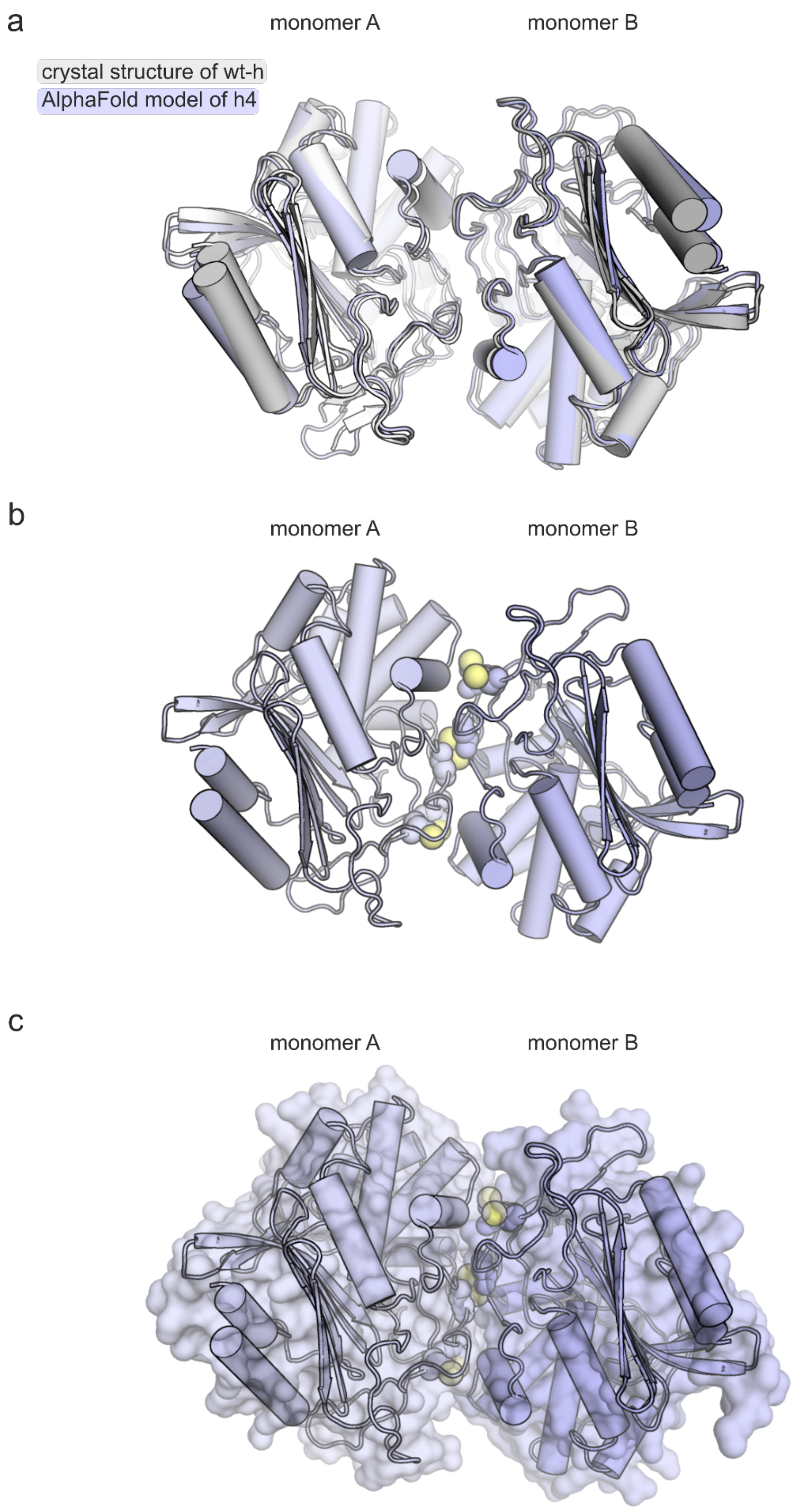

Supporting Figure S21: a) Overlay of the wt-h crystal structure (grey, PDB ID 9spl) with the h4 dimer AlphaFold model (blue). b) AlphaFold model of the h4 dimer in cartoon representation showing the INCYPRO cysteines as spheres (sulfur atoms in yellow). c) Same structure as is Figure S21b including transparent surface representation.

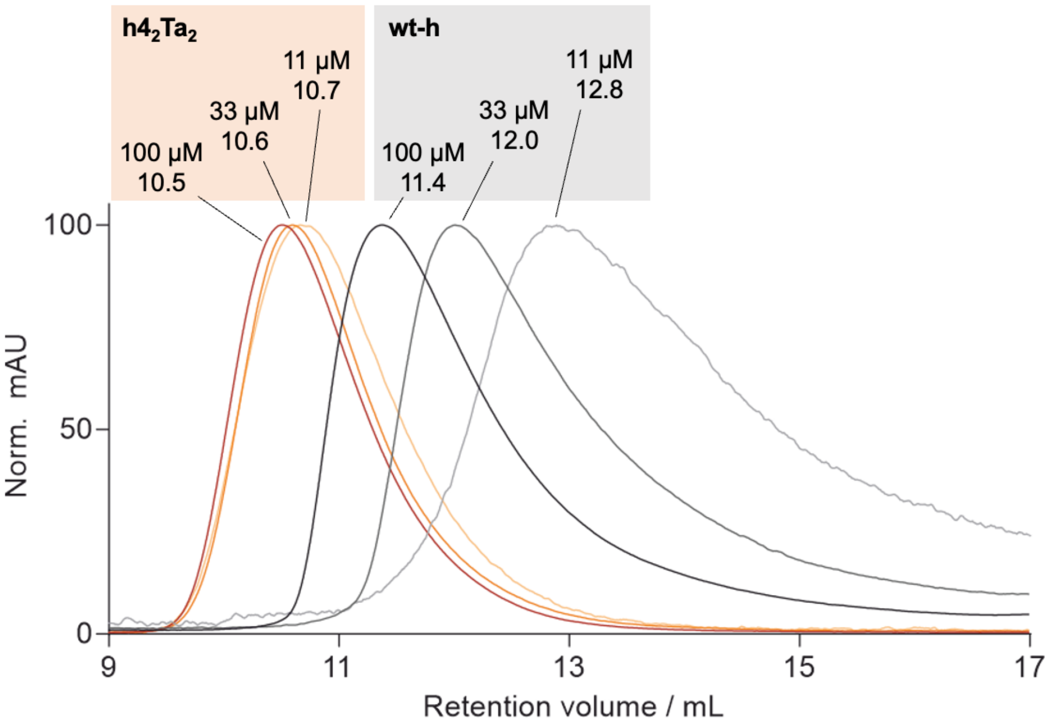

Supporting Figure S22: Size exclusion chromatography of wt-h (grey) and $h4_2Ta_2$ (orange) at different concentrations including the corresponding retention volumes in mL. Proteins are measured at 100, 33 and 11 µM in 50 mM HEPES pH 8.0, 50 mM NaCl using an ÄKTA Pure system (Cytiva) equipped with a Superdex 75 Increase 10/300 GL column. The column was equilibrated with 50 mM HEPES pH 8.0, 50 mM NaCl at 4 °C. Protein samples (200 µL) were injected and eluted at a flow rate of 0.5 mL/min.

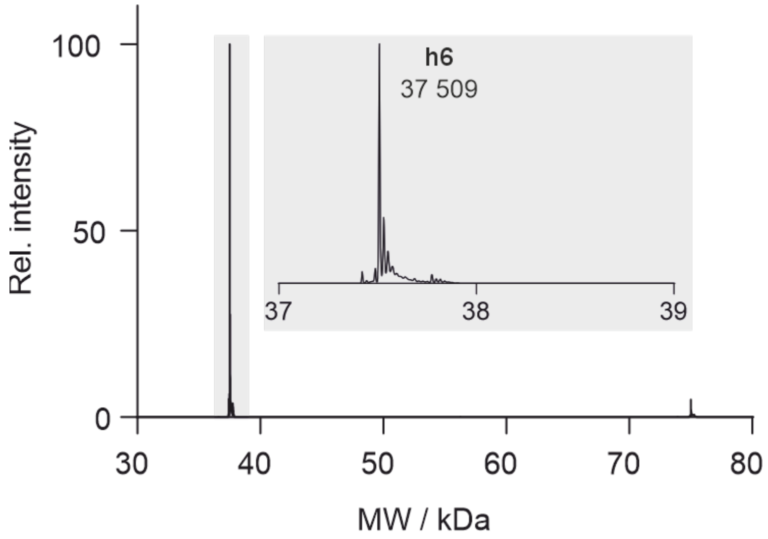

Supporting Figure S23: Deconvoluted TOF-MS spectra of h6 (calc. MW = 37506 Da). Data collected on Agilent LC/MSD XT ESI-Quadrupole LC-MS. For ESI-MS spectrum and peak list, see Supporting Figure S24.

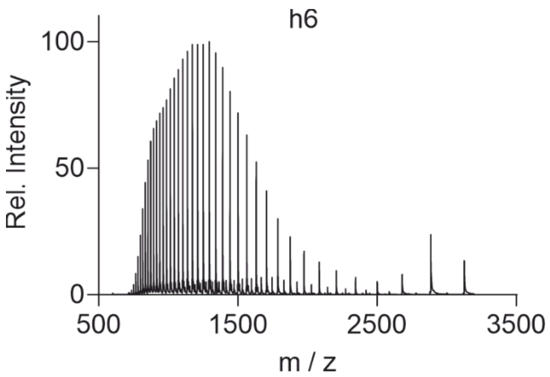

h6 (D11C/K36C/K347C/K136C/N205C/Y232C)
calc.: 37505.6 Da
found: 37509.2 Da

h6		
**Charge**	**m/z found $[M+H]^+$**	**m/z calc.**
50	782.5	782.4
49	799.1	799.0
48	816.4	816.3
47	834.6	834.5
46	853.5	853.4
45	873.3	873.2
44	894.1	894.0
43	915.9	915.8
42	938.8	938.6
41	962.8	962.7
40	988.1	988.0
39	1014.8	1014.7
38	1042.9	1042.8
37	1072.7	1072.6
36	1104.2	1104.1
35	1137.7	1137.5
34	1173.2	1173.1
33	1211.0	1210.9
32	1251.3	1251.2
31	1294.4	1294.3
30	1340.6	1340.5
29	1390.2	1390.1
28	1443.6	1443.5
27	1501.3	1501.2

Supporting Figure S24: ESI-MS spectrum and peak list for h6. Data collected on Agilent LC/MSD XT ESI-Quadrupole LC-MS.

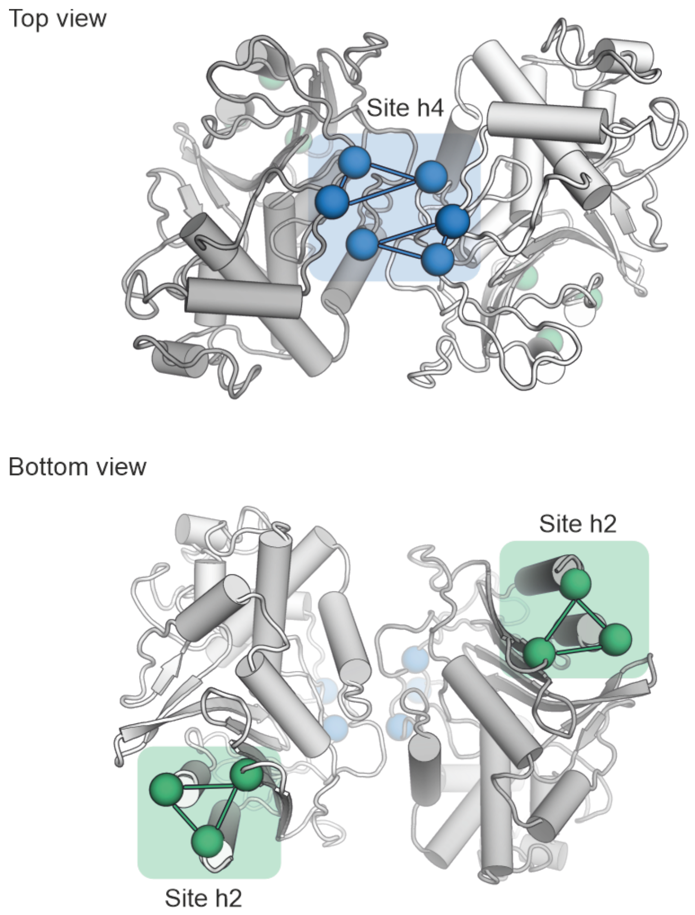

Supporting Figure S25: Model of INCYPRO sites h2 (green) and h4 (blue) in protein h6. The crosslinking pattern of the two adjacent h4 sites (blue) has been confirmed by protein crystallography of $h4_2Ta_2$ (manuscript Figure 4).

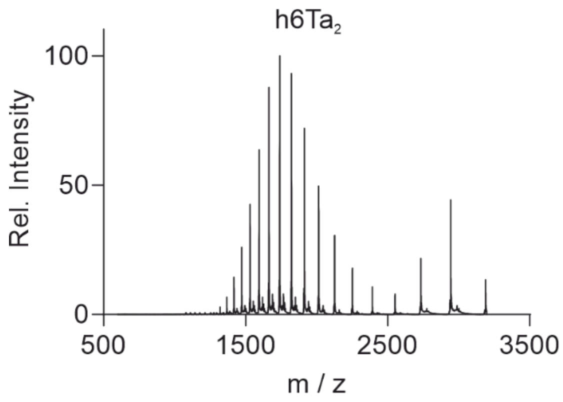

h6Ta₂
calc.: 38261.9 Da
found: 38262.8 Da

h6Ta₂		
**Charge**	**m/z found [M+H]⁺**	**m/z calc.**
28	1367.5	1367.6
27	1418.1	1418.2
26	1472.6	1472.7
25	1531.5	1531.5
24	1595.3	1595.3
23	1664.6	1664.6
22	1740.2	1740.2
21	1823.0	1823.0
20	1914.1	1914.1
19	2014.8	2014.8
18	2126.7	2126.7
17	2251.7	2251.7
16	2392.4	2392.4
15	2551.8	2551.8
14	2734.0	2734.0
13	2944.2	2944.3
12	3189.5	3189.5

Supporting Figure S26: ESI-MS spectrum and peak list for h6Ta₂. Data collected on Agilent LC/MSD XT ESI-Quadrupole LC-MS.

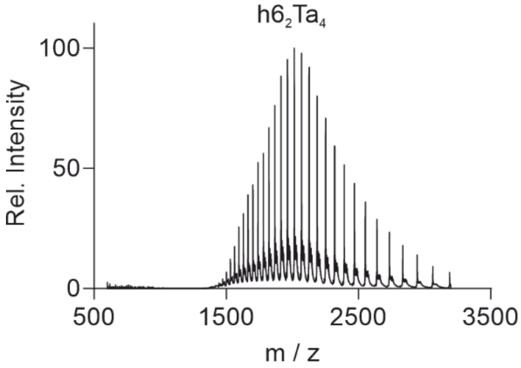

h6$_2$Ta$_4$
calc.: 76523.8 Da
found: 76525.4 Da

h6$_2$Ta$_4$		
**Charge**	**m/z found [M+H]$^+$**	**m/z calc.**
49	1562.9	1562.7
48	1595.4	1595.3
47	1629.3	1629.2
46	1664.8	1664.6
45	1701.7	1701.5
44	1740.3	1740.2
43	1780.8	1780.6
42	1823.1	1823.0
41	1867.6	1867.4
40	1914.2	1914.1
39	1963.3	1963.2
38	2014.9	2014.8
37	2069.3	2069.2
36	2126.8	2126.7
35	2187.5	2187.4
34	2251.8	2251.7
33	2320.0	2319.9
32	2392.5	2392.4
31	2469.6	2469.5
30	2551.9	2551.8
29	2639.9	2639.8
28	2734.1	2734.0
27	2835.3	2835.2
26	2944.4	2944.2

Supporting Figure S27: ESI-MS spectrum and peak list for h6$_2$Ta$_4$. Data collected on Agilent LC/MSD XT ESI-Quadrupole LC-MS.

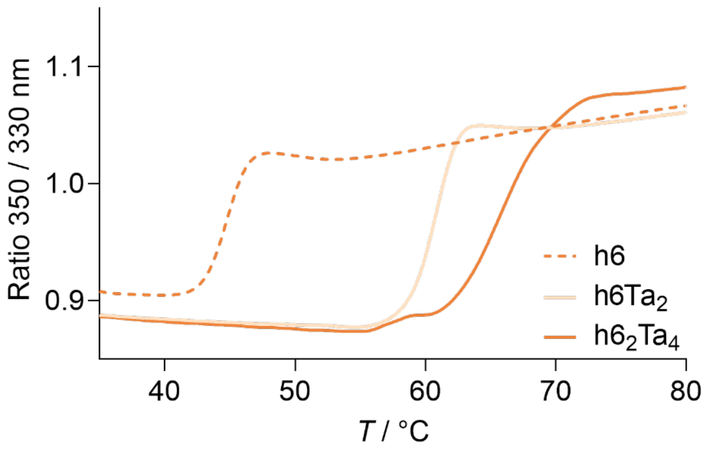

Supporting Figure S28 DSF thermal denaturation curves of h6, h6Ta$_2$ and h6$_2$Ta$_4$ after optimization of the cross-linking reaction. Measured at 50 µM monomer concentration (in 50 mM HEPES pH 8.0, 50 mM NaCl).

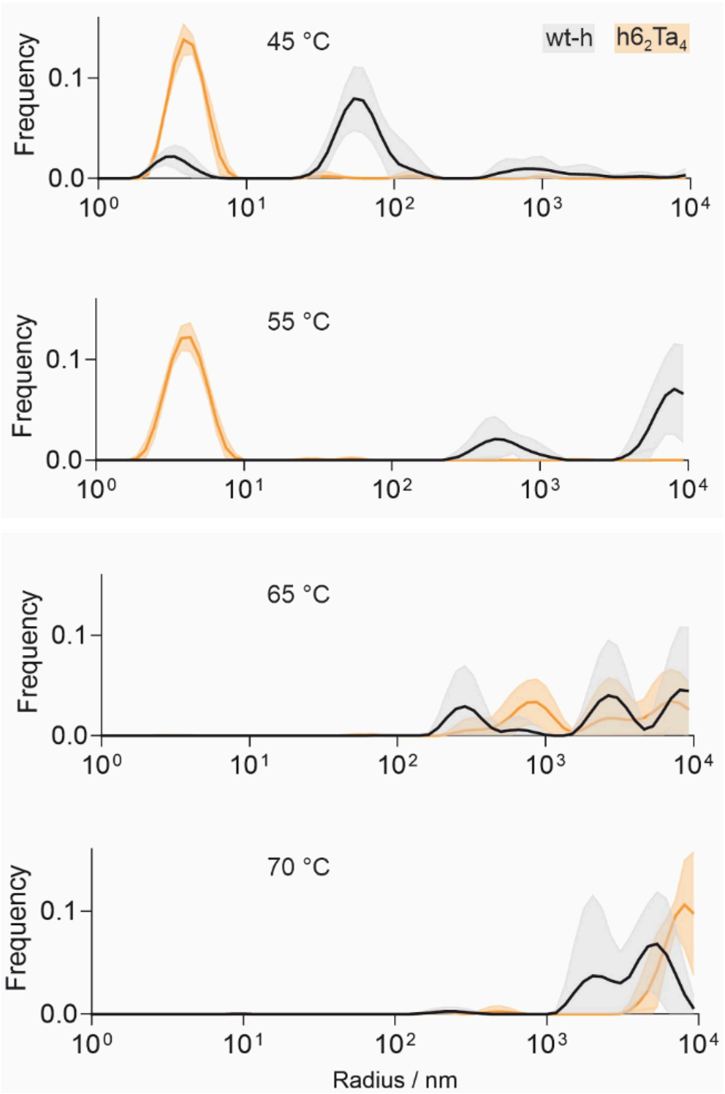

Supporting Figure S29: Temperature-dependent size distribution derived from DLS experiment for wt-h (grey) and h6$_2$Ta$_4$ (orange). Measured at a 20 µM (wt-h) or 50 µM (h6$_2$Ta$_4$) monomer concentration (in 50 mM HEPES pH 8.0, 50 mM NaCl). The errors account for 1σ (n = 5).

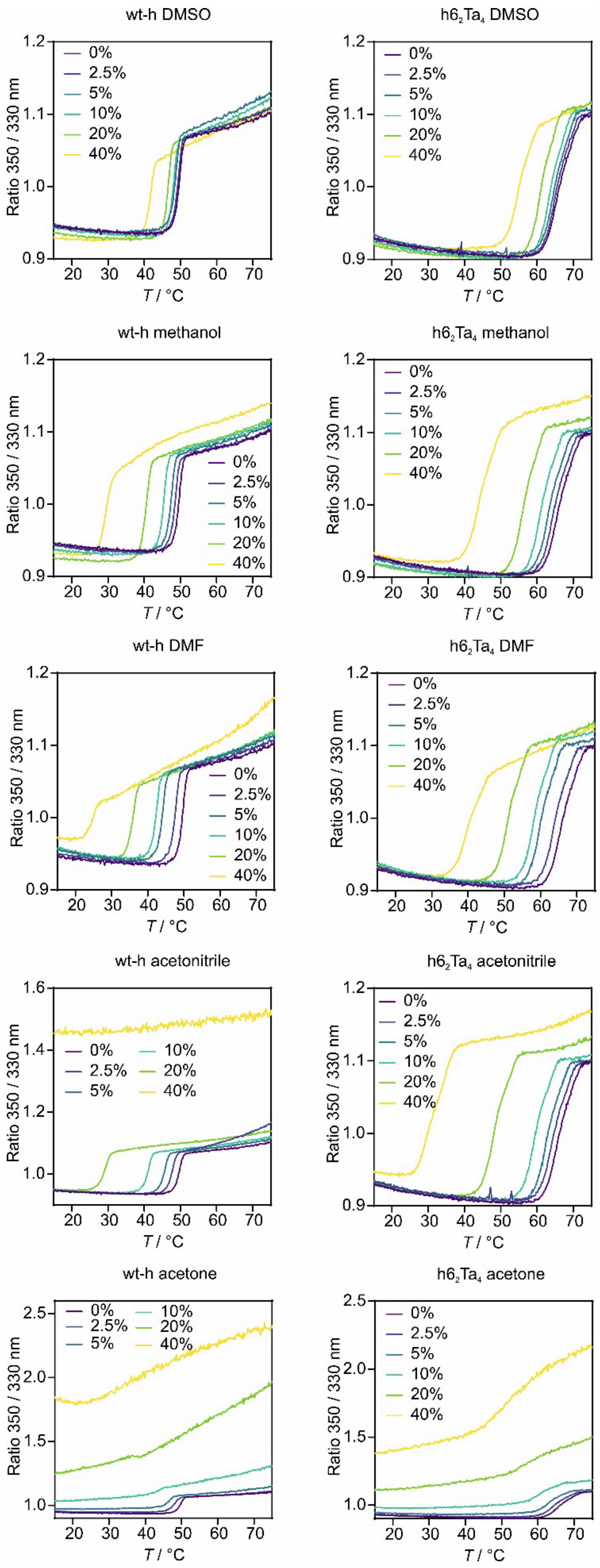

Supporting Figure S30: Thermal denaturation profiles of wt-h and h6$_2$Ta$_4$ in presence of varying concentrations of DMSO, methanol, DMF, acetonitrile and acetone. Measured at a 10 µM monomer concentration (in 50 mM HEPES pH 8.0, 50 mM NaCl).

a

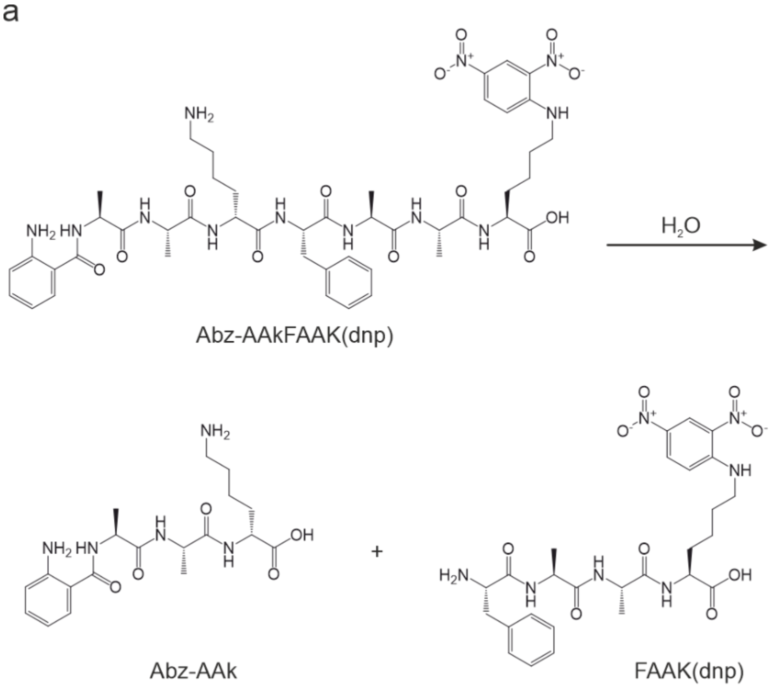

b

t / min	wt-h	$h6_2Ta_4$
0	100.1 ±1.6	100.0 ±2.8
5	0.1 ±0.1	—
30	-0.1 ±0.2	—
270	—	89.4 ±4.2
960	—	68.3 ±1.6
1380	—	61.4 ±8.3
4020	—	32.7 ±4.5

c

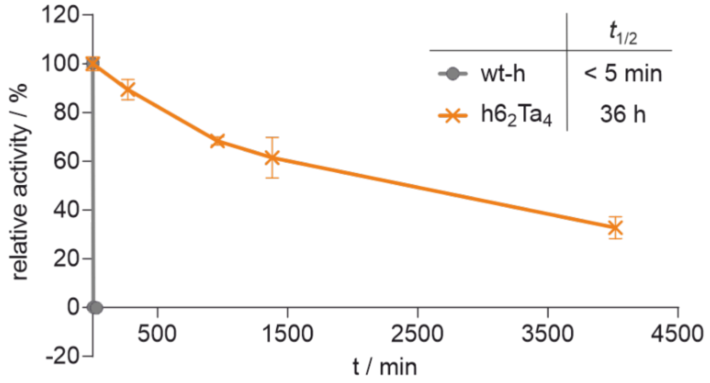

Supporting Figure S31: Time-dependent relative enzymatic activity of wt-h and $h6_2Ta_4$ at 50 °C in the presence of 10% ethanol. a) Chemical structure of the peptide substrate (Abz-AAkFAAK(dnp)) used in the measurements (Abz = 2-amino benzoic acid, dnp = 2,4-dinitrophenol). b) Table with percentage of relative enzymatic activity at different time points (errors = 1σ, $n \geq 3$). c) Corresponding plots of time-dependent enzymatic activity of wt-h and $h6_2Ta_4$ at 50 °C in presence of 10% ethanol including estimated half-life values ($t_{1/2}$). An excitation wavelength of 320 nm and an emission wavelength of 420 nm were used (50 µM substrate, 100 mM phosphate buffer pH 8.0, 150 mM NaCl and 0.5 µM enzyme).

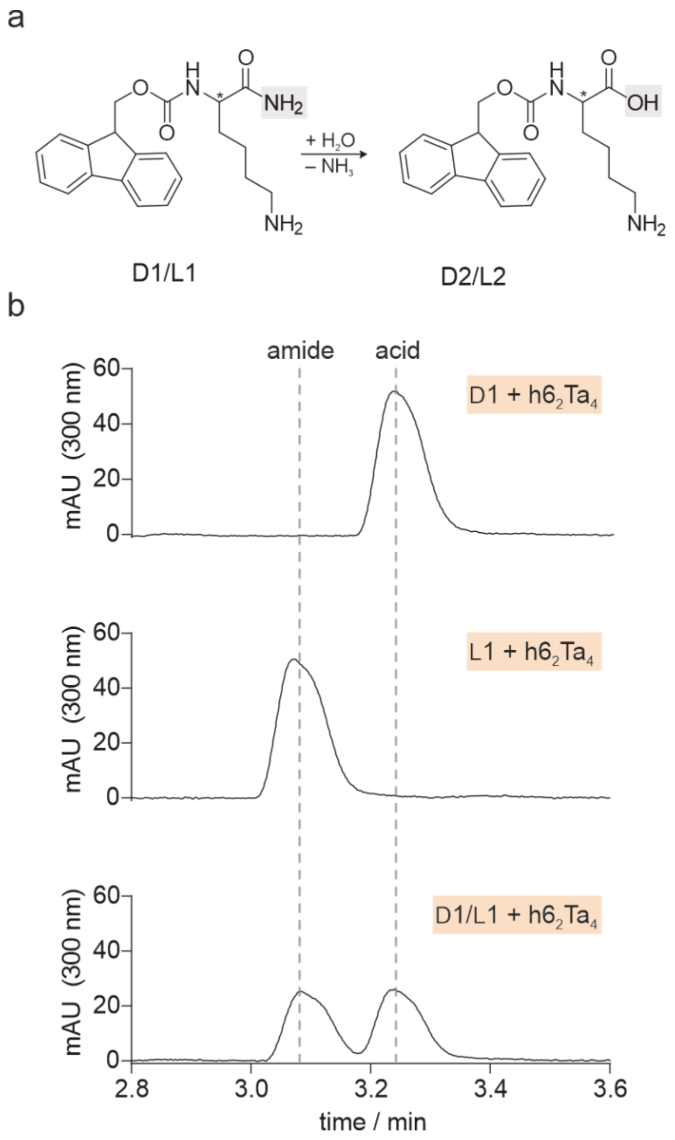

Supporting Figure S32: a) Scheme of the hydrolysis of the D- and L-configured substrate. b) Chiral UPLC separation of enantiomers and the racemate using 50 µM of the substrate (Fmoc-k/K-NH_2, D1/L1, T = 50 °C in 10% ethanol, UPLC readout at λ = 300 nm) in 100 mM phosphate buffer pH 8.0, 150 mM NaCl. D/L (bottom) refers an equimolar mixture of the D- and the L-substrate with a final concentration of 50 µM.

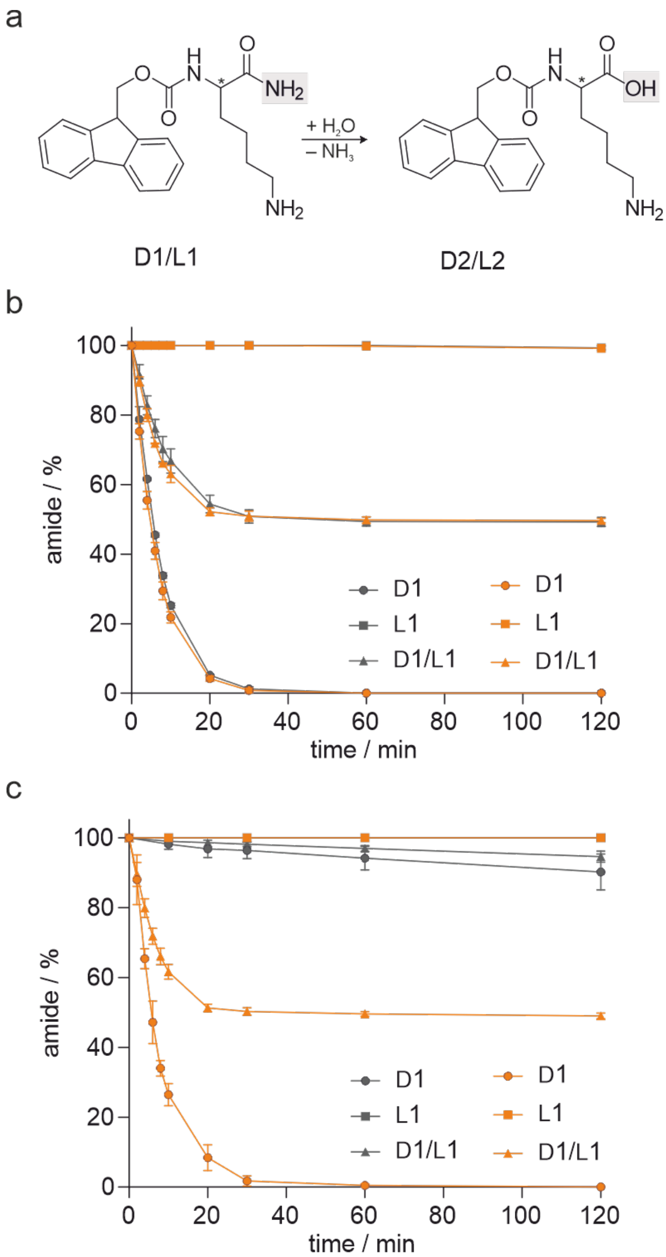

d

protein	D			L		
wt-h	25	19	10	0	0	0
h6$_2$Ta$_4$	749	863	671	0	0	0

Supporting Figure S33: Time-dependent conversion of enantiomers as well as racemic mixtures of Fmoc-k/K-NH_2 (c = 50 µM) using wt-h (grey) and h6$_2$Ta$_4$ (orange). Readout was performed via UPLC (λ = 300 nm) and measured in 100 mM phosphate buffer pH 8.0, 150 mM NaCl at a 0.1 µM monomer concentration and 50 µM amide substrate concentration). a) Scheme of the hydrolysis of the D- and L-configured substrate. b) Enzymatic reaction at 30°C. Considering the detection limit of this method, the enantiomeric excess (ee) of the enzyme-generated product is >99 % in both cases. c) Enzymatic reaction at 50 °C in the presence of 10% EtOH. For values see Supporting Table S6 and S7. d) Overview of enzyme activity ($A_{s,app}$) in mkatal mol^{-1} for wt-h and h6$_2$Ta$_4$ at 50 °C in the presence of 10% EtOH.

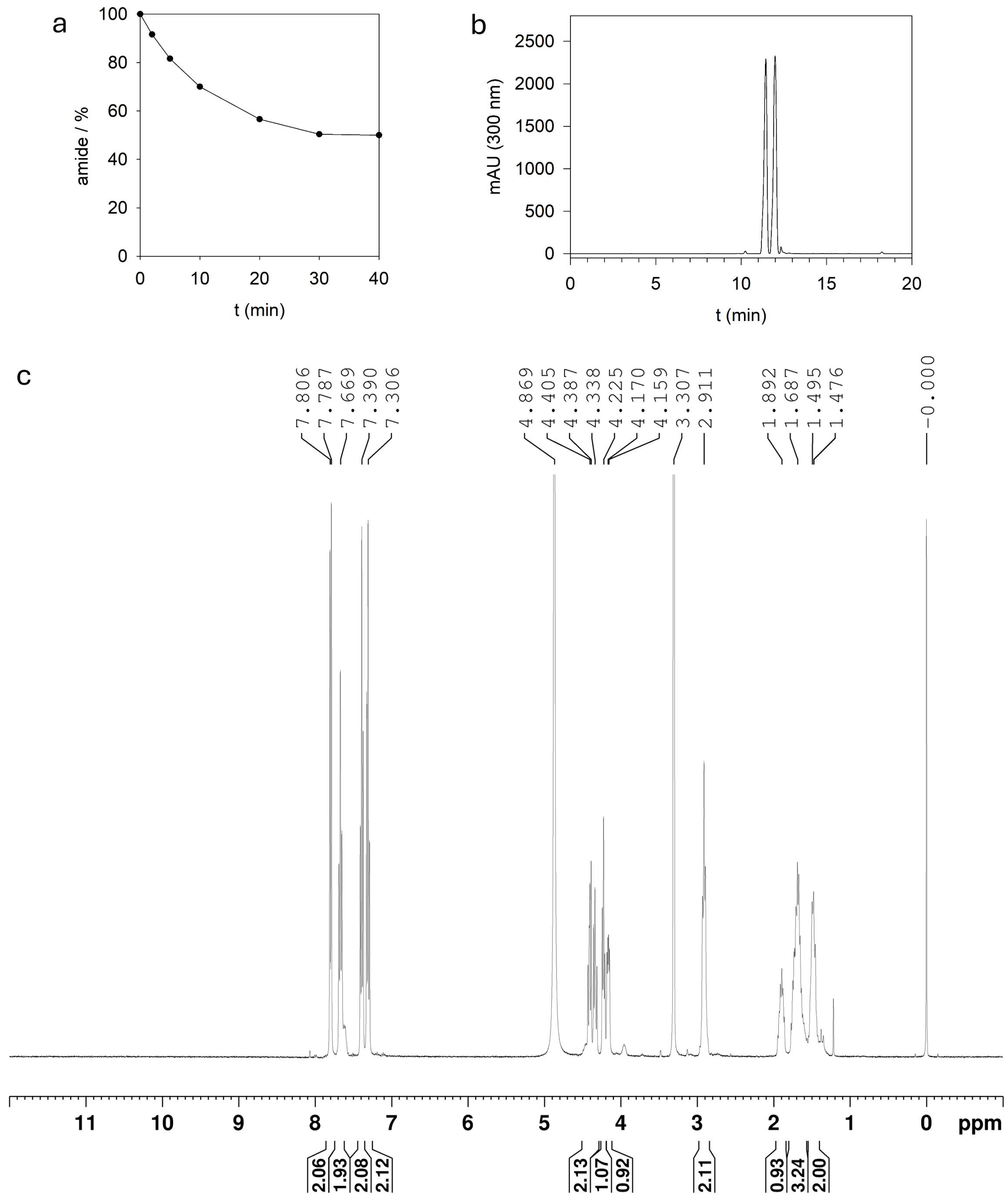

Supporting Figure S34: Enzymatic resolution of racemate in preparative scale. Hydrolase activity of h6$_2$Ta$_4$ in a racemic mixture of Fmoc-k/K-NH$_2$ at 50 °C in 10% ethanol in a final volume of 15 mL. a) Time course of amide hydrolysis (readout with UPLC at 300 nm, c_{enzyme} = 0.1 µM, based on monomer, $c_{\text{Fmoc-lysine}}$ = 2 mM, buffer: 100 mM phosphate pH 8.0, 150 mM NaCl). b) HPLC profile of 5 mL reaction mixture after 40 min incubation time using a XSelect® Peptide CSH C18, 10 mm x 250 mm. Elution was performed with an acetonitrile/water gradient from 5 to 90% (v/v) acetonitrile containing 0.1% trifluoracetic acid at a flow rate of 3 mL/min. Peak 2 of three runs were collected (retention time = 12.0 min), lyophilized and analyzed via NMR. c) NMR analysis of isolated product Fmoc-D-Lys-OH ^{1}H-NMR (400 MHz, MeOD) δ = 7.80 (d, *J* = 7.5 Hz, 2H), 7.67 (t, *J* = 7.9 Hz, 2H), 7.39 (t, *J* = 7.5 Hz, 2H), 7.31 (t, *J* = 7.4 Hz, 2H), 4.44-4.30 (m, 2H), 4.22 (t, *J* = 6.8 Hz, 1H), 4.19–4.13 (m, 1H), 2.91 (t, *J* = 7.3 Hz, 2H), 1.96–1.85 (m, 1H), 1.78–1.60 (m, 3H), 1.54–1.42 (m, 2H).

Supporting Tables

Protein	Variations	Cα-Cα distances / Å		
h1	K36C<br>V296C<br>L316C	V296–L316<br>9.6	L316–K36<br>12.3	K36–V296<br>14.3
h2	D11C<br>K36C<br>K347C	D11–K347<br>7.1	K347–K36<br>10.1	K36–D11<br>11.2
h3	K57C<br>K213C<br>D247C	K213–K57<br>8.5	K57–D247<br>12.6	D247–K213<br>13.6
h4	K136C<br>N205C<br>Y232C	Y232–K136<br>8.5	K136–N205<br>10.3	N205–Y232<br>11.2
h5	T140C<br>S182C<br>N205C	N205–<br>T140<br>9.1	T140–S182<br>13.0	S182–N205<br>13.3

Supporting Table S1: Distances between the Cα atoms of selected cross-linking sites in DHy wt-h.

Structure	DHy (PDB ID: 9s7k)
Data collection	DLS, I04
detector	DECTRIS EIGER 2 XE 16M
wavelength (Å)	0.9537Å
resolution limits (Å)	43.12–1.77 (1.97–1.77)
space group (#)	*P1* (1)
cell dimensions	
a, b, c (Å)	53.78, 53.76, 57.37
α, β, γ (°)	75.69, 75.69, 87.81
# total reflections	245954 (11736)
# unique reflections	35659 (1783)
multiplicity	6.9 (6.6)
completeness (spherical) (%)	60.8 (11.3)
completeness (ellipsoidal) (%)	91.8 (56.7)
I / σI	11.8 (1.4)
$CC_{1/2}$	0.999 (0.490)
R_{pim}	0.034 (0.509)
Refinement	
# total reflections	35648 (142)
# reflections R_{free}	1751 (7)
R_{work} / R_{free}	0.240 / 0.270
# atoms (non-hydrogen)	5406
protein	5194
ligand	54
water	158
protein residues	678
r.m.s. deviations	
bond lengths (Å)	0.0024
angles (°)	0.8860
ramachandran	
favored regions / %	96.4
allowed regions / %	3.3
outliers / %	0.3
Rotamer outliers / %	1.2
Clashscore	2.4
MolProbity score	1.32
B-factor (average)	43.7
protein	43.9
ligand	62.8
water	30.1

Supporting Table S2: Crystallographic table of the data collection and refinement data of the crystal structure of $h4_2Ta_2$ (PDB ID 9s7k). Values in parenthesis correspond to the highest resolution shell.

T / °C	wt-h	h2Ta	h4Ta	$h4_2Ta_2$	$h6Ta_2$	$h6_2Ta_4$
30	9.8 ±0.6	11.3 ±0.5	7.4 ±0.8	10.3 ±0.6	7.6 ±1.5	11.0 ±0.3
40	20.6 ±1.1	24.7 ±1.6	13.0 ±0.3	21.3 ±1.2	15.3 ±0.6	22.9 ±1.0
50	29.6 ±2.4	40.8 ±1.8	21.8 ±1.8	40.2 ±3.3	24.0 ±2.6	41.0 ±2.3
55	3.4 ±1.0	42.5 ±1.9	18.3 ±1.8	38.8 ±1.3	23.5 ±1.7	45.5 ±1.9
60	0.03 ±0.03	30.1 ±3.7	0.4 ±0.2	15.2 ±0.8	21.3 ±1.8	47.1 ±1.6
65	0.0 ±0.0	0.0 ±0.0	0.0 ±0.0	0.0 ±0.0	11.7 ±2.1	30.0 ±1.0
70	0.0 ±0.0	0.0 ±0.0	0.0 ±0.0	0.0 ±0.0	0.0 ±0.0	3.5 ±0.9
75	0.0 ±0.0	0.0 ±0.0	0.0 ±0.0	0.0 ±0.0	0.0 ±0.0	0.0 ±0.0

Supporting Table S3: Enzyme activity ($A_{s,app}$) in mkatal mol^{-1} for wt-h, h2Ta, h4Ta, $h4_2Ta_2$, $h6Ta_2$ and $h6_2Ta_4$ (UPLC 360 nm readout, measured in 100 mM Pi pH 8.0, 150 mM NaCl at 0.5 µM monomer protein concentration and 50 µM substrate). The errors account for 1σ ($n \geq 3$).

% solvent	0	2.5	5	10	20	40
**wt-h**						
DMSO	49.4	49.2	48.5	48.0	46.5	41.6
Methanol	49.4	48.5	47.3	45.3	40.5	29.5
Ethanol	49.4	47.7	46.1	43.2	36.6	no T_m
DMF	49.4	47.5	44.0	42.3	35.8	24.7
Acetonitrile	49.4	47.7	45.5	41	29.1	no T_m
Acetone	49.4	47.9	46.4	no T_m	no T_m	no T_m
**h6$_2$Ta$_4$**						
DMSO	65.8	65.2	64.7	63.6	61.1	55.0
Methanol	65.8	64.5	63.2	61.0	56.2	44.1
Ethanol	65.8	64.0	62.2	58.6	50.7	37.0
DMF	65.8	63.8	60.0	58.3	51.0	39.7
Acetonitrile	65.8	64.3	62.7	59.3	48.1	30.4
Acetone	65.8	64.3	63.0	60.4	no T_m	no T_m
**ΔT_m**						
DMSO	16	16	16	16	15	13
Methanol	16	16	16	16	16	15
Ethanol	16	16	16	15	14	wt-h no T_m
DMF	16	16	16	16	15	15
Acetonitrile	16	17	17	18	19	wt-h no T_m
Acetone	16	16	17	wt-h no T_m	—	—

Supporting Table S4: Melting temperatures of wt-h and h6$_2$Ta$_4$ in presence of different cosolvents derived from the thermal denaturation profiles. Measured at a 10 µM monomer concentration (in 50 mM HEPES pH 8.0, 50 mM NaCl). The melting temperatures are reported in °C.

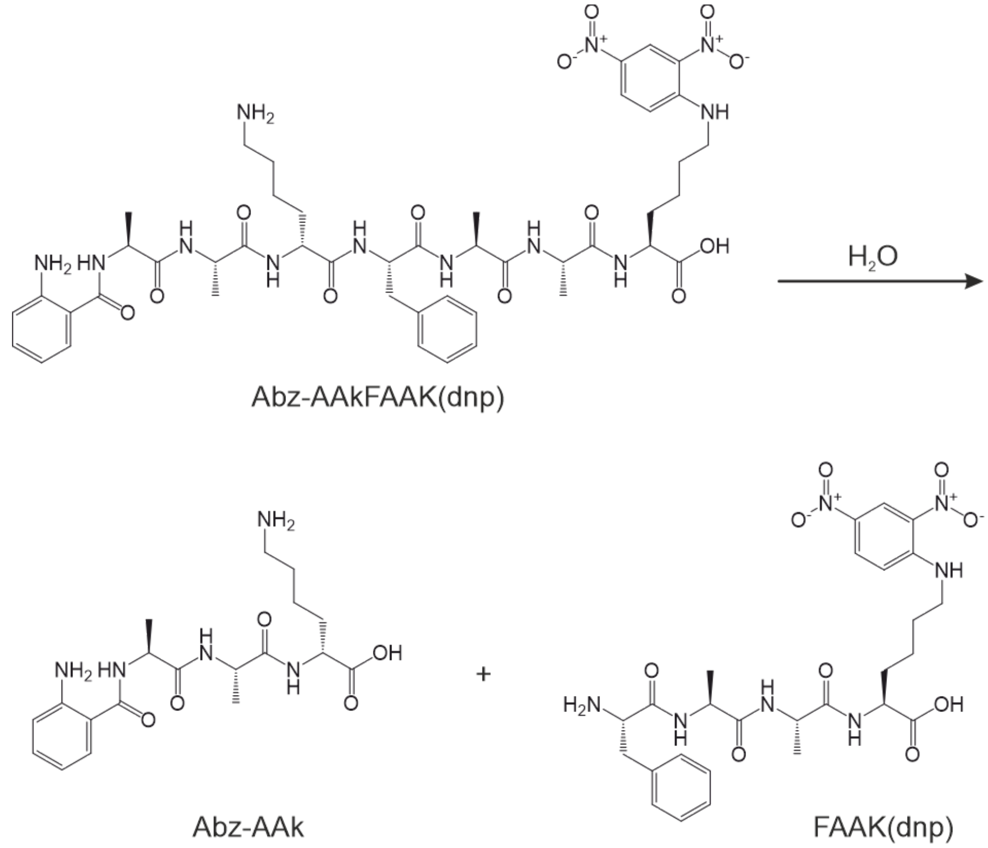

[EtOH] / %	wt-h	h6$_2$Ta$_4$
0	30 ±2	41 ±2
1	19 ±1	30 ±2
2.5	11 ±1	24 ±1
5	2.1 ±0.5	15 ±2
7.5	<0.1	9.2 ±0.4
10	<0.1	5.4 ±0.8
15	<0.1	1.4 ±0.1
20	<0.1	<0.1
25	<0.1	<0.1

Supporting Table S5: Enzymatic activity ($A_{s,app}$) in mkatal mol^{-1} of wt-h and h6$_2$Ta$_4$ at 50°C in the presence of varying amounts of ethanol (UPLC 360 nm readout, measured in 100 mM Pi pH 8.0, 150 mM NaCl at 0.5–1.0 µM monomer protein concentration and 50 µM substrate). The errors account for 1σ ($n \geq 3$).

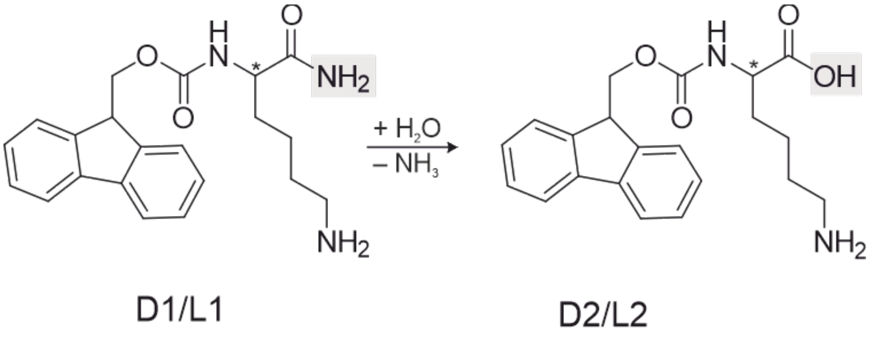

t / min	wt-h			$h6_2Ta_4$		
	D1	L1	D1/L1	D1	L1	D1/L1
0	100 ±0.0	100 ±0.0	100 ±0.0	100 ±0.0	100 ±0.0	100 ±0.0
2	78.7 ±3.7	100 ±0.0	91.5 ±3.0	75.3 ±2.2	100 ±0.0	89.6 ±1.3
4	61.6 ±0.5	100 ±0.0	82.8 ±2.7	55.5 ±2.6	100 ±0.0	80.0 ±1.8
6	45.5 ±0.6	100 ±0.0	76.2 ±2.6	40.9 ±2.4	100 ±0.0	71.8 ±0.1
8	33.8 ±0.8	100 ±0.0	70.2 ±3.6	29.4 ±2.5	100 ±0.0	66.0 ±0.1
10	25.3 ±0.7	100 ±0.0	66.8 ±3.5	21.8 ±1.6	100 ±0.0	63.0 ±2.5
20	5.2 ±0.2	100 ±0.0	54.4 ±2.5	4.2 ±0.8	100 ±0.0	52.2 ±1.1
30	1.2 ±0.7	100 ±0.0	50.8 ±1.9	0.8 ±0.7	100 ±0.0	50.9 ±1.5
60	0.0 ±0.0	100 ±0.0	49.4 ±1.3	0.05 ±0.1	99.8 ±0.3	49.8 ±0.9
120	0.0 ±0.0	99.3 ±0.6	49.3 ±1.4	0.03 ±0.1	99.2 ±0.0	49.7 ±0.7

Supporting Table S6: Substrate conversion in the presence of wt-h and $h6_2Ta_4$, respectively, using 50 µM Fmoc-k/K-NH_2 as substrate at 30 °C (UPLC 300 nm readout, measured in 100 mM phosphate buffer pH 8.0, 150 mM NaCl at a 0.1 µM monomer concentration and 50 µM amide substrate concentration) D1/L2 refers to an equimolar mixture of the D- and the L-substrate with a final concentration of 50 µM. The errors account for 1σ (*n* ≥ 3).

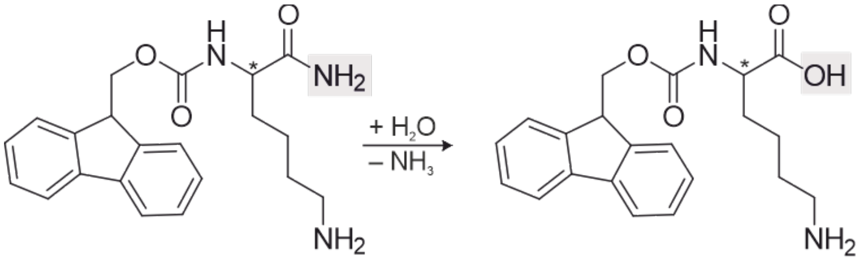

t / min	wt-h			$h6_2Ta_4$		
	D1	L1	D1/L1	D1	L1	D1/L1
0	100 ±0.0	100 ±0.0	100 ±0.0	100 ±0.0	100 ±0.0	100 ±0.0
2	—	—	—	88.0 ±7.1	—	89.6 ±3.5
4	—	—	—	65.4 ±2.9	—	79.9 ±2.6
6	—	—	—	47.2 ±6.1	—	71.8 ±2.3
8	—	—	—	34.0 ±2.3	—	66.0 ±2.4
10	98.1 ±1.5	100 ±0.0	99.0 ±0.3	26.4 ±3.2	100 ±0.0	61.7 ±2.1
20	96.9 ±2.5	—	98.6 ±0.03	8.4 ±3.7	—	51.3 ±1.1
30	96.4 ±2.4	100 ±0.0	98.2 ±0.6	1.7 ±1.5	100 ±0.0	50.3 ±1.1
60	94.2 ±3.4	100 ±0.0	97.0 ±0.8	0.5 ±0.4	100 ±0.0	49.6 ±0.7
120	90.3 ±5.2	100 ±0.0	94.6 ±1.6	0.0 ±0.0	100 ±0.0	49.0 ±0.8

Supporting Table S7: Substrate conversion in the presence of wt-h and $h6_2Ta_4$, respectively, using 50 µM Fmoc-k/K-NH_2 as substrate at 50 °C in 10% ethanol (UPLC 300 nm readout, measured in 100 mM phosphate buffer pH 8.0, 150 mM NaCl at a 0.1 µM monomer concentration and 50 µM amide substrate concentration) D1/L2 refers to an equimolar mixture of the D- and the L-substrate with a final concentration of 50 µM. The errors account for 1σ (*n* ≥ 3).

References

[1] M. R. Wilkins, E. Gasteiger, A. Bairoch, J.-C. Sanchez, K. L. Williams, R. D. Appel, D. F. Hochstrasser in *2- Proteome Anal. Protoc.*, Humana Press, New Jersey, **1998**, pp. 531–552.

[2] F. Frottin, A. Martinez, P. Peynot, S. Mitra, R. C. Holz, C. Giglione, T. Meinnel, "The Proteomics of N-terminal Methionine Cleavage" *Mol. Cell. Proteomics* **2006**, *5*, 2336–2349.

[3] F. Gorrec, "The MORPHEUS protein crystallization screen" *J. Appl. Crystallogr.* **2009**, *42*, 1035–1042.

[4] W. Kabsch, "*XDS*" *Acta Crystallogr. D Biol. Crystallogr.* **2010**, *66*, 125–132.

[5] C. Vonrhein, C. Flensburg, P. Keller, A. Sharff, O. Smart, W. Paciorek, T. Womack, G. Bricogne, "Data processing and analysis with the *autoPROC* toolbox" *Acta Crystallogr. D Biol. Crystallogr.* **2011**, *67*, 293–302.

[6] E. Krissinel, A. A. Lebedev, V. Uski, C. B. Ballard, R. M. Keegan, O. Kovalevskiy, R. A. Nicholls, N. S. Pannu, P. Skubák, J. Berrisford, M. Fando, B. Lohkamp, M. Wojdyr, A. J. Simpkin, J. M. H. Thomas, C. Oliver, C. Vonrhein, G. Chojnowski, A. Basle, A. Purkiss, M. N. Isupov, S. McNicholas, E. Lowe, J. Triviño, K. Cowtan, J. Agirre, D. J. Rigden, I. Uson, V. Lamzin, I. Tews, G. Bricogne, A. G. W. Leslie, D. G. Brown, "*CCP* 4 Cloud for structure determination and project management in macromolecular crystallography" *Acta Crystallogr. Sect. Struct. Biol.* **2022**, *78*, 1079–1089.

[7] A. Vagin, A. Lebedev, "*MoRDa* , an automatic molecular replacement pipeline" *Acta Crystallogr. Sect. Found. Adv.* **2015**, *71*, s19–s19.

[8] P. Emsley, K. Cowtan, "*Coot* : model-building tools for molecular graphics" *Acta Crystallogr. D Biol. Crystallogr.* **2004**, *60*, 2126–2132.

[9] K. Yamashita, M. Wojdyr, F. Long, R. A. Nicholls, G. N. Murshudov, "*GEMMI* and *Servalcat* restrain *REFMAC* 5" *Acta Crystallogr. Sect. Struct. Biol.* **2023**, *79*, 368–373.
